# New miRNAs cloned from neuroblastoma

**DOI:** 10.1186/1471-2164-9-52

**Published:** 2008-01-29

**Authors:** Elena A Afanasyeva, Agnes Hotz-Wagenblatt, Karl-Heinz Glatting, Frank Westermann

**Affiliations:** 1 Department of Tumour Genetics, B030, German Cancer Research Center, Im Neuenheimer Feld 280, D-69120 Heidelberg, Germany; 2 HUSAR Bioinformatics Laboratory, Department of Molecular Biophysics, B020, German Cancer Research Center, Im Neuenheimer Feld 280, D-69120, Heidelberg, Germany

## Abstract

**Background:**

MicroRNAs (miRNAs) are a novel class of gene expression regulators implicated in cancer biology. Neuroblastoma (NB) is an embryonal tumour consisting of neural crest-derived undifferentiated cells and is characterised by variable clinical courses ranging from spontaneous regression to therapy-resistant progression. Recent advances identified a subset of miRNAs with putative function in NB biology. However, the full repertoire of miRNAs expressed in NBs is not available.

**Results:**

We describe miRNA profiles of 13 NB specimens and 2 NB cell lines as determined by miRNA cloning. A total of 3153 sequences were sequenced and analysed by a miRNA prediction tool (miRpredict). Our library covered 27% miRNAs known to date. 39 reads corresponding to 25 individual sequences were classified as novel miRNAs, including miRNA* species of 10 known miRNAs. Expression of 5 new miRNA* forms and 8 individual sequences was supported by Northern blotting. Most of the novel miRNA genes are not related to each other and do not share homology with the annotated sequences in the public miRNA database, but they are conserved within mammals or have close homologues in primates genomes.

**Conclusion:**

We provide evidence for 29 new miRNA and miRNA-like sequences (24 novel sequences and 5 miRNAs discovered initially in other species). Some of these newly identified sequences reside within frequently altered chromosomal regions in NB tumours and may play a role in NB biology.

## Background

Neuroblastoma is the second commonest solid cancer in young children accounting for 9% of all childhood cancers. It is characterised by a heterogeneous clinical behaviour ranging from spontaneous regression in 10% of all cases to rapid progression with unfavourable prognosis [1,2]. Amplified *MYCN *leading to high *MYCN *mRNA and protein levels plays an important role in NB biology and is used as a powerful prognostic marker in NB risk stratification. In addition, several other genetic abnormalities, including gain of 17q, 11q and 1p deletion have been associated with an aggressive NB phenotype [3]. Further, microarray technology has been used to study gene expression profiles in primary NBs. Patterns of differentially expressed genes among different NB subtypes as well as gene expression classifiers have emerged allowing a better prediction of patient's outcome than established risk markers [4,5].

MiRNAs that regulate gene expression at the posttranscriptional level have been described to play a role in carcinogenesis via executing oncogenic or tumour suppressive functions [6-8]. For example, *let-7 *miRNAs are down-regulated in lung cancer and are known to target proto-oncogene RAS transcripts [9]. Mir-17-92 cistron is to be overexpressed in human lung cancer and upregulated by c-Myc [10,11]. *miR-15 *and *miR-16 *are frequently deleted and down-regulated in chronic lymphocytic leukemias [12]. The expression profiles of a small set of miRNAs could be used to classify different types of cancer [13-15]. More recently, profiling of a subset of known miRNAs in neuroblastoma specimens suggested that MYCN acts as a regulator of miRNAs [16].

However, the full repertoire of miRNAs expressed in different cancer types, including neuroblastoma, is not yet available. Prognosis-relevant miRNAs with putative oncogenic functions could have been missed in previous cloning efforts due to their silent state or low-expression. There is evidence to date that neural lineage cells are particularly rich in miRNA diversity [17-19]. In this study, we aimed at cloning novel miRNAs from NB cell lines and NB specimens that may have a role in NB biology. Here, we present the analysis of small RNA libraries derived from neuroblastoma tumour specimens and cell lines and suggest several novel miRNAs. Some of these miRNAs reside within loci of cancer-associated rearrangements and are of special interest for future studies.

## Results and discussion

### MiRNA cloning and abundance of previously annotated miRNAs

For NB miRNA library construction, we used RNA derived from 13 tumour samples as well as two neuroblastoma cell lines, SH-EP (*MYCN *single-copy) and Kelly (*MYCN *amplified). We followed the guidelines of Lau et al. ensuring that only those RNA species were cloned which contained 5'-phosphate [20]. The following cohort of tumours was used for cloning: four *MYCN *amplified, three stage 4S *MYCN *single-copy, two stage 4 *MYCN *single-copy and four stage 1 *MYCN *single-copy tumours (Table 1).

**Table 1:** MYCN status and stage of tumours

**TUMOUR**	STAGE*	**MYCNamplification**
MYCNAMP_NB1	1	+
MYCNAMP_NB2	3	+
MYCNAMP_NB3	3	+
MYCNAMP_NB4	4	+
MYCNSC_NB1	1	-
MYCNSC_NB2	1	-
MYCNSC_NB3	1	-
MYCNSC_NB4	1	-
MYCNSC_NB5	4S	-
MYCNSC_NB6	4	-
MYCNSC_NB7	4S	-
MYCNSC_NB8	4S	-
MYCNSC_NB9	4	-

* NB stage was evaluated according to INSS criteria

Approximately 80 clones per specimen or cell line were sequenced. After linker unmasking, 3185 small RNA cDNA sequences were subjected to further analyses. The percentage of "not-known-miRNA" sequences varied from 18% to 60% which corresponds to previously described fractions of other non-coding RNAs, redundant sequences as well as novel miRNAs in small RNA libraries [17,21]. Our cloning project covered 27% (128 miRNAs) of miRNAs currently present in the public miRNA registry (miRBase 9.1) [22]. 39 miRNAs were represented by only one read. On the other hand, mir-124a (with three loci on 8p23.1, 8q12.3, 20q13.33) and -125b (11q24.1 and 21q21.1) yielded 113 and 132 reads, respectively (see Additional File 1). MiRNA cloning was established as an independent semi-quantitative method to evaluate miRNA abundance [23]. This allowed us to conclude that mir-124a and -125b were the most abundant ones. Both miRNAs are considered as having neuronal specificity [24,25]. Among the most frequently identified miRNAs was mir-21 (17q23.2), a transcript with putative anti-apoptotic and tumour promoting activities [8]. A difference in the abundance of certain miRNAs was observed in *MYCN*-amplified and non-amplified specimens. In *MYCN*-amplified tumours/cell line, we observed a higher abundance of miRNAs from the mir-17/92 cluster (13q31.3) that is known to be up-regulated by c-Myc. The average numbers for hsa-mir-17-5p and hsa-mir-20a reads in *MYCN*-amplified and single-copy specimens were 5 *versus *0.8 and 3.6 *versus *0.6, respectively. A group of miRNA sequences that clustered on chromosome 19 was exclusively found in the library of the *MYCN *amplified tumour, MYCNAMP_NB2. This argues for a cistronic manner of transcription of this group of miRNA sequences. Beside annotated human miRNAs we have cloned 5 sequences that have been originally identified in rat and mouse (see Additional File 1).

### Identification and validation of new miRNAs

After removing redundant sequences and the sequences derived from other non-coding RNAs, the remaining pool was checked *in silico *for the detection of the putative miRNA precursors using miRPredict (see Methods). This analysis yielded 25 distinct sequences (39 reads) which were classified as miRNAs. Of these 25 miRNAs, 10 sequences were miRNA* forms of previously annotated miRNA precursors (Table 2). Hsa-mir-594 was cloned by the Velculescu group, but then excluded from miRBase due to overlap with a tRNA gene [26]. Cloning of the 594* species described here provides evidence for the miRNA nature of these sequences.

**Table 2:** Novel human miRNA* sequences identified by cloning from NB

**Sequence name**	**Sequence**	**Chromosomal location**	**Northern blot validation**	**Reads**	**Notes**
HSA-MIR-188*	ctcccacatgcagggtttgca	Xp11.22	-	1	3'-arm
HSA-MIR-106b*	ccgcactgtgggtacttgctgc	7q22.1	+	5	3'-arm
HSA-MIR-423*	tgaggggcagagagcgaga	17q11.2	+	1	5'-arm
HSA-MIR-594*	cctaagccagggattgtgggtt	7q34	-	1	5'-arm
HSA-MIR-342*	aggggtgctatctgtgattgagg	11q32.2	+	1	5'-arm
HSA-MIR-490*	ccatggatctccaggtgggt	7q33	-	2	5'-arm
HSA-MIR-361*	cccccaggtgtgattctgatttg	Xq21.1	+	3	3'-arm
HSA-MIR-28*	cactagattgtgagctcctgga	3q28	-	1	3'-arm
HSA-MIR-127*	ctgaagctcagagggctctgat	14q32.31	-	1	5'-arm
HSA-MIR-214*	tgcctgtctacacttgctgtgca	1q24.3	+	2	5'-arm

Notes give the information which hairpin arm expresses the miRNA* form.

**Figure 1:**
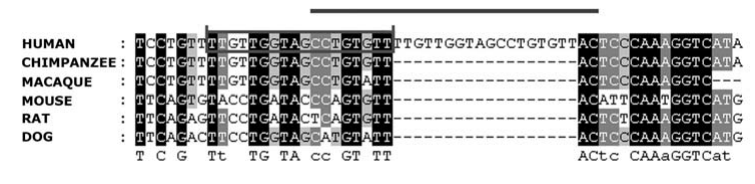
MYCNAMP_NB4_70, a novel miRNA-like sequence resulting from a duplication event. Short sequences flanking MYCNAMP_NB4_70 were extracted from the human genome, as well as homologuous sequences from chimpanzee, macaque, mouse, rat and dog genomes and aligned. The bar indicates the MYCNAMP_NB4_70 sequence. The duplication is marked by a box.

**Figure 2:**
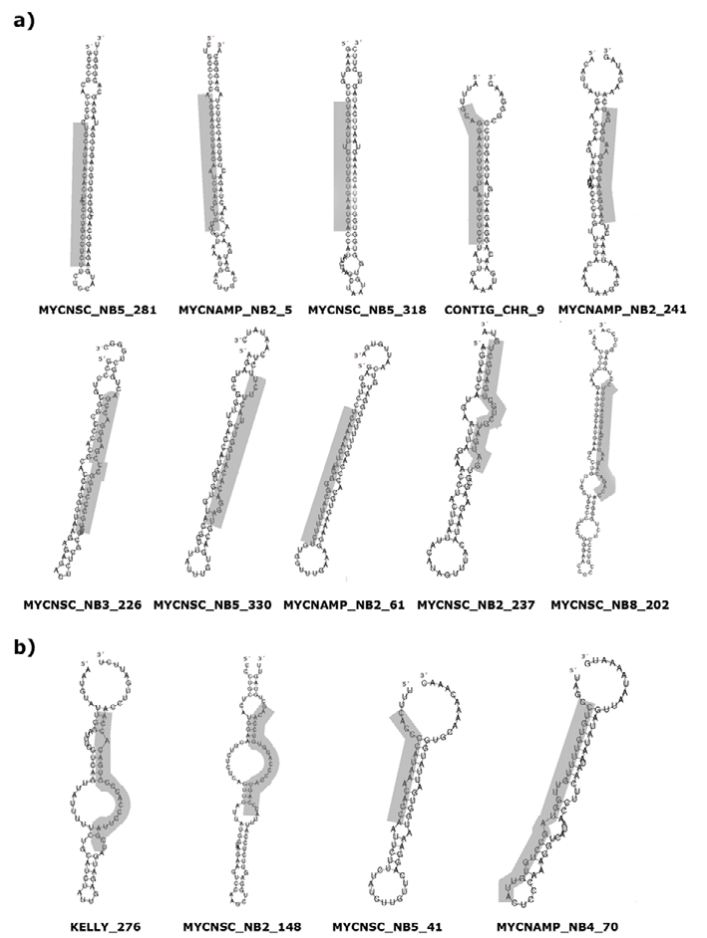
Predicted secondary structures of the putative novel human miRNA precursors. Human genomic sequences upstream and downstream of the novel miRNAs were folded with the computer program RNAfold. Grey areas represent the cloned miRNA. a) miRNA with canonical hairpin. b) borderline structures.

Other 15 sequences represent novel small RNAs which have not been annotated. Two novel sequences were found adjacent to chromosomal localisations of known miRNAs: MYCNSC_NB5_330 within a miRNA cluster on chromosome 14 as well as MYCNAMP_NB2_61 within a miRNA cluster on chromosome 19 (Table 3). Phylogenetic conservation was determined for each putative miRNA and its surrounding with respect to chimpanzee, macaque, opossum, mouse, rat, dog, bull and chicken genomes (see Additional File 2). We found that the cloned sequences with the exception of MYCNAMP_NB4_70 are conserved either within all chosen species or primates. The non-conserved MYCNAMP_NB4_70 sequence seems to be the result of a short duplication within an intron of the DPP10 transcript (Fig. 1). However, homologous regions in chimpanzee and macaque genomes are also able to form hairpins which might give rise to miRNAs. Novel miRNAs do not share any homology with each other and therefore do not comprise a family. Searching against miRBase revealed that none of the new miRNAs are related to the annotated miRNAs in miRBase, except of one sequence, MYCNAMP_NB2_5, that is homologous to hsa-mir-151 and -28. Analysis of genomic locations of the individual miRNAs showed that 5 of them are localized in extragenic regions. 8 sequences are found within introns of coding transcripts. MYCNSC_NB5_64, classified as a miRNA by miRpredict, is found within a predicted U5 gene which reduces reliability of this sequence as a miRNA (Table 3). The likely precursors of the novel miRNAs (Fig. 2) may be subdivided into two subgroups: structures with typical hairpin or borderline precursors. The latter subgroup has features divergent from a canonical miRNA hairpin, such as bulges (KELLY_276, MYCNSC_NB2_148), short stem (MYCNSC_NB5_41) or "oscillating" hairpin (MYCNAMP_NB4_70), where the candidate miRNA can reside on the 5' or 3'-arm. Lui et al. reported the cloning of a subset of non-canonical miRNAs, however the relevance of such RNAs to the classical miRNA pathway remains to be determined [27].

**Table 3:** Novel human miRNA sequences identified by cloning from NB

**Sequence name**	**Sequence**	**Chromosomal location**	**Northern blot validation**	**Reads**	**Notes**
MYCNAMP_NB2_5	aaggagcttacaatctagctggg	11q14.1	+	1	predicted gene
MYCNSC_NB2_148	acccagcaccccaggtttccacag	12q13.12	-	1	Tubulin-K-alpha-1
MYCNSC_NB5_330	taggacacatggtctacttct	14q32.31	+	1	within miRNA cluster
MYCNAMP_NB2_61	tcaaaactgaggggcattttct	19q13.42	+	4	within miRNA cluster
MYCNAMP_NB4_70	cctgtgttttgttggtagcctgtgttac	2q14.1	-	1	DPP10
MYCNSC_NB8_202	ccagacagaattctatgcactttc	3p12.3	-	1	KIAA0664
MYCNSC_NB5_41	tcaccccataaacacca	3p13	+	1	Extragenic
MYCNSC_NB5_281	tccattacactaccctgcctct	3p25.3	-	1	Plasma membrane calcium ATPase isoform 2
KELLY_276	tcgattcccacccctgacacca	4q13.1	+	1	Extragenic
MYCNSC_NB3_226	ctgccctggcccgagggaccga	5q31.2	-	1	Kelch-like-3
CONTIG_CHR_9	tgcaggaacttgtgagtctcc	9p21.1	+	4	Extragenic
MYCNSC_NB5_318	tggatttctttgtgaatca	9p21.1	+	1	Extragenic
MYCNAMP_NB2_241	cagggaggtgaatgtgat	Xq21.2	-	1	Extragenic
MYCNSC_NB5_64	aagctaattttttgaggcc	15q22.31	NA	1	U5 predicted, borderline case
MYCNSC_NB2_237	gatgatgctgctgatgctg	8p23.1	+	1	Pin2-interacting protein X1

Notes include the information about genomic locations and additional sequence information.

**Figure 3:**
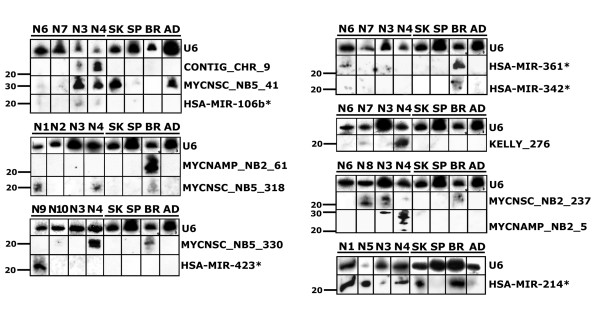
Northern blot analysis of newly cloned miRNAs. N1...N10 – tumour samples; BR-Brain, AD-Adrenal gland, SK-Skeletal muscle, SP-Spleen. The positions of marker are indicated. U6 RNA was used as a loading control.

Based on the data listed above, we subjected 24 new miRNAs to Northern blot validation using biotinylated probes. Borderline case MYCNSC_NB5_64 sequences were not further examined. The expression of each putative miRNA was checked in a panel of human tissues, including different tumour specimens, normal brain, adrenal gland, spleen and skeletal muscle tissues. 5 of 10 cloned miRNA* sequences and 8 sequences from individual hairpins were Northern blot positive (Fig. 3). The other 11 sequences remained undetectable (data not shown). The probe for MYCNSC_NB5_41 yielded a signal around 30 nt which is higher than the estimated sequence size. The probes for CONTIG_CHR_9, MYCNSC_NB5_237 and MYCNAMP_NB2_5 yielded several bands different in length by 1–3 nucleotides. Some known miRNAs show such patterns in Northern blot analyses and intermediate products of miRNA maturation have been observed, but there is no comprehensive explanation for these results [19,28,29]. In normal tissues, Northern blot-positive miRNAs are preferentially expressed in brain, skeletal muscle tissue and adrenal gland, being less frequently expressed in spleen. The detection of miRNAs in skeletal muscle might be due to the presence of motor neurons. Several miRNAs showed differential expression in normal versus tumour specimens: CONTIG_CHR_9, KELLY_276, MYCNAMP_NB2_5 were expressed in tumours, but not in normal tissues. On the other hand, we did not find MYCNAMP_NB2_61 miRNA in tumour specimens, but we found strong expression of this sequence in brain.

## Conclusion

Recently, several studies have been performed aiming at directional cloning of tissue/tumour specific miRNAs [13,26,27]. The importance of such an approach is highlighted by the fact that patterns of novel miRNAs from tumours of different lineages hardly overlap and may include miRNAs with a functional role in tumourigenesis. Our study provides evidence for 29 new human miRNAs cloned from neuroblastoma. 5 of them were originally cloned from other species and have not been annotated in the human miRNA database. 10 sequences represent miRNA* species of previously annotated sequences. 14 sequences, which have not been annotated before, are suggested to be novel miRNAs. Some of these miRNAs are found within regions of chromosomal rearrangements associated with cancer. For example, new miRNAs on 9p21 residing within NB-related aberration region are of special interest for future studies [30]. miRNA profiling of these novel sequences will allow us to clarify their relevance in NB development, progression and regression.

## Methods

### Tumour specimens and cell culture

SH-EP and Kelly cells were cultured in RPMI supplemented with 10% fetal calf serum, glutamate and penicillin/streptomycin. NB tumour samples were collected prior to any cytoreductive treatment and were frozen immediately until RNA was isolated. Written informed consent was obtained from patients' parents for tissue sampling. Genomic *MYCN *status was assessed in the reference laboratories of the German NB trial in Köln and Heidelberg.

### Small-RNA library construction and sequencing

Small-RNA fractions were directly isolated with the use of the mirVana miRNA Isolation Kit (Ambion). 18–26 nt RNA library construction was performed as described by Lau and coworkers [20]. Sequencing was performed by GATC BIOTECH (Konstanz, Germany).

### Sequence analysis and prediction of novel miRNAs

Quality and vector trimming of sequences were performed with Vector NTI software. After unmasking linker sequences, inserts of ≥17 bp were analysed using miRPredict, a miRNA prediction pipeline [31]. miRPredict compares each potential miRNA to both known miRNAs and to other non-coding RNAs (EnsEMBL RNA database, Assembly 36) with a pattern-match algorithm using the EMBOSS program fuzznuc [32]. Sequences corresponding to other non-coding RNAs were discarded and the rest was localised in the genome via Blast search. The genomic hit together with additional 50 bases to both sides were extracted as potential precursor miRNA. In case that more than one localization in a contig was found, as indicated by equal score and expectation values of the BLAST search, all localizations were used for further processing. In the precursor DNA, palindromic structures are recognized by the program PALINDROME (EMBOSS package, allowing 4 mismatches in the stem, loop max. 40 bases, length between 15 and 50). If a palindrome was found, the palindromic region was folded with RNAFOLD (Vienna package) to get the energy value and the input file for the triplet SVM classifier [33,34]. The triplet SVM determines the fold as miRNA-like palindrome (value +1), other palindrome (value -1) or regions with more than one loop (empty output).

To assess hairpin conservation, we extracted alignments of putative miRNA precursors from chimpanzee, macaque, mouse, rat, dog, cow, opossum and chicken genomes via EnsEMBL. Extracted sequences were checked for hairpin structures with the use of RNAfold, and aligned by RNAforester [35].

### Northern blotting

Total RNA was isolated with TRIZOL reagent (Invitrogen). 30 micrograms of total RNA was loaded per lane and separated on 15% denaturing polyacrylamide gels, transferred by electroblotting to GeneScreen+ membranes (Perkin Elmer). To check for non-radioactive probe specificity, 250 pg of synthetic 5'-phosphorylated sequence identical to the respective miRNA was loaded along with 5'-phosphorylated marker sequences (20 + 30 nt, 250 pg each, see Additional Files 3 and 4). Blots were hybridized overnight at 37°C with radioactively α-(^32^P) labeled DNA oligo probes complementary to the cloned sequences in Church buffer, washed twice with 2 × SSC, 0.1% SDS at 37°C, and exposed to films (Fuji Film). For non-radioactive detection, blots were incubated with 3'-biotinylated probes (10 ng/ml) and then processed with North2 South Chemiluminescent Hybridization and Detection Kit (Pierce). Prolonged washing was applied after incubation with HRP-streptavidine conjugate.

## Authors' contributions

FW designed the study. EAA constructed libraries, performed validation and drafted the manuscript. AHW, KHG and EAA performed analysis. All authors read and approved the final manuscript.

## Supplementary Material

Additional file 1 Composition of miRNA libraries from neuroblastoma. Known miRNAs were counted in each of the libraries and in total. Click here for file

Additional file 2 Assessment of phylogenetic conservation of novel miRNAs. Putative precursor sequences of the novel miRNAs were extracted from the human genome along with the respective homologous sequences from other genomes and aligned. The secondary structures of the precursor RNAs are included as determined by RNAfold. Click here for file

Additional file 3 Method of comparison of biotinylated probes. Northern blot procedure for comparison of biotinylated probes is outlined. The file also contains the description of Additional File 4. Click here for file

Additional file 4 Comparison of biotinylated probes for the detection of miRNAs. Results of Northern blotting comparing the probes used to validate novel miRNAs. Click here for file
