# Supplementary material for: New miRNAs cloned from neuroblastoma
Source: BMC Genomics. 2008 Jan 29;9:52. doi: 10.1186/1471-2164-9-52 (PMC2254388; doi:10.1186/1471-2164-9-52)
Supplement: Additional file 2 — Assessment of phylogenetic conservation of novel miRNAs. Putative precursor sequences of the novel miRNAs were extracted from the human genome along with the respective homologous sequences from other genomes and aligned. The secondary structures of the precursor RNAs are included as determined by RNAfold. [file 1471-2164-9-52-S2.doc]

------------------------------------------------------------------------------------------------------------------------------------------------------------------------------------------------

--------------

MYCNSC_NB8_202

--------------

Identity: Ptr 0,98 Mml 0,50 Mdo 0,41 Bta 0,62

CCAGACAGAATTCTATGCACTTTC

MYCNSC_NB8_202_Hsa ----------AGGTGCATGAAGCCTGGTCCTGC-CCTCACTGGGAACCCCCT-TCCCTCTGGGTA**CCAGACAGAATTCTATGCACTTTC**CTGGAGGCTCCATGCTGGTCTGTTCATTTGGAAGTTTGAGGCTGTCCATGAGGA-

MYCNSC_NB8_202_Ptr ----------AGGTGCATGAAGCCTGGTCCTGC-CCTCACTGGGAACCCCCT-TCCCTCTGGGTA**CCAGACAGAATTCTGTGCACTTTC**CTGGAAGCTCCATGCTGGTCTGTTCATTTGGAAGTTTGAGGCTGTCCATGAGGA-

MYCNSC_NB8_202_Mml ATTCCTGGACAGGTGC---TACCCAGACCCGCC--CTTCCTGC---CAGGCT-CATATGCACCTC**CCAGTAGGCTCCCTCTG**-----**TT**CTCTAGGGGCC--TATCCACAGGGAAGT-GGAAGAAGGGGATAACCCAG------

MYCNSC_NB8_202_Mdo ATTCCTGGCCAGGTGCCC-TGCCAGCCCCCTTCTCCCCTCAGCCTCCTGGGTACTTCTCCAAAGC**CCTTTCACATTCATGTC**---**TGTT**CTCTTTG---CAGTGCCCTCATGGCACAAGGGAGGGTGGGAGGCTCATTTCAAAG

MYCNSC_NB8_202_Bta ATTCCTGGACAGGTGCCTGTGGCCATCCCCGGCTCCTCTCT------CCCTT-CCCCTGCGGGGC**CCAGGCTGGGTTCTCGG**----**TGC**CTGTAGG------GCTGCACAGGGTGGCAGGG-GGGTGGGACTGCCCCG------

MYCNSC_NB8_202_Hsa

AGGUGCAUGAAGCCUGGUCCUGCCCUCACUGGGAACCCCCUUCCCUCUGGGUA**CCAGACAGAAUUCUAUGCACUUUC**CUGGAGGCUCCAUGCUGGUCUGUUCAUUUGGAAGUUUGAGGCUGUCCAUGAGGA

.((((((((((..(((....(((((.....(((((.....)))))...))))).....)))..))).)))))))(((.(((((((..((.(((..((((......))))))).))..))).)))).))).. (-43.20)

MYCNSC_NB8_202_Ptr

AGGUGCAUGAAGCCUGGUCCUGCCCUCACUGGGAACCCCCUUCCCUCUGGGUA**CCAGACAGAAUUCUGUGCACUUUC**CUGGAAGCUCCAUGCUGGUCUGUUCAUUUGGAAGUUUGAGGCUGUCCAUGAGGA

.((((((((((..(((....(((((.....(((((.....)))))...))))).....)))..))).)))))))(((.(((((((..((.(((..((((......))))))).))..))).)))).))).. (-43.10)

MYCNSC_NB8_202_Mml

AUUCCUGGACAGGUGCUACCCAGACCCGCCCUUCCUGCCAGGCUCAUAUGCACCUC**CCAGUAGGCUCCCUCUGUU**CUCUAGGGGCCUAUCCACAGGGAAGUGGAAGAAGGGGAUAACCCAG

...(((((.((((.((...........))....)))))))))........(((.((((.((((((.((((.........)))))))))).....)))).)))......(((.....))).. (-41.60)

MYCNSC_NB8_202_Mdo

AUUCCUGGCCAGGUGCCCUGCCAGCCCCCUUCUCCCCUCAGCCUCCUGGGUACUUCUCCAAAGC**CCUUUCACAUUCAUGUCUGUU**CUCUUUGCAGUGCCCUCAUGGCACAAGGGAGGGUGGGAGGCUCAUUUCAAAG

......(((.(((...)))))).(((((((((((((....(((....(((((((....(((((.......(((....))).......))))).)))))))....)))....))))))).))).)))........... (-47.14)

MYCNSC_NB8_202_Bta

AUUCCUGGACAGGUGCCUGUGGCCAUCCCCGGCUCCUCUCUCCCUUCCCCUGCGGGGC**CCAGGCUGGGUUCUCGGUGC**CUGUAGGGCUGCACAGGGUGGCAGGGGGGUGGGACUGCCCCG

........((((....))))(((..((((..((((((((((((((..((((((((((((.(((......))).))).)))))))))......)))).)).))))))))))))..)))... (-52.00)

MYCNSC_NB8_202_Hsa .((((((((((..(((....(((((.....(((((.....)))))...)))))**.....)))..))).)))))))**------**(((**---.((((----(((---..--((.(-

MYCNSC_NB8_202_Ptr .((((((((((..(((....(((((.....(((((.....)))))...))))).....**)))..))).)))))))**------**(((**---.((((----(((---..--((.(-

MYCNSC_NB8_202_Mml .--.---.-(--((((.---((((-.----((...........))....)))))-----)))--)..-......------(((---.(**((**-------**(**---**.(**--**((((**-

MYCNSC_NB8_202_Mdo .--.---.-.--..-(----((.(------((.-----.---.))--)--)-))--------------------.(((-((((--((((((....(((....(((((((.

MYCNSC_NB8_202_Bta .--.---.-.--....----((-(------(..-----.---.-)--)--)-)---------------------(((..((((..(((((((((-(((-((..--((((-

MYCNSC_NB8_202_Hsa ---((..--((-(--(.-.-....))-)--)---)-)-).))-.-----.)))----.))))-.)-))..-----------------

MYCNSC_NB8_202_Ptr ---((..--((-(--(.-.-....))-)--)---)-)-).))-.-----.)))----.))))-.)-))..-----------------

MYCNSC_NB8_202_Mml ---**(.(**---**((**-**(**--....-.....)-)--)---)-)-))))-)-----....----.))))-.)-))......(((.....)))..

MYCNSC_NB8_202_Mdo ...(((((.**......(((....))).....**..))))).))))))-)....)))-....))))))).))).)))..---.........

MYCNSC_NB8_202_Bta ---(((((((**(.(**--**((......))).))).**-**)**))))-))))......)))).)).)))))))))-)))..))).-------.---.

------------------------------------------------------------------------------------------------------------------------------------------------------------------------------------------------

---------------

MYCNAMP_NB2_241

---------------

Identity: Ptr 0,98 Mmu 0,51 Rno 0,48

CAGGGAGGTGAATGTGAT

MYCNAMP_NB2_241_Hsa ACATTATGAAGCAAGTATTATTATCCC-TGTTTTACAAATAAGGAAATAAACT**CAGGGAGGTGAATGTGAT**CAAAGATAGATATATAGTTAAGAAAGTTAAAGAGACAGGATTCAAGCCTAAGTCTGTCTG-

MYCNAMP_NB2_241_Ptr ACATTATGAAGCAAGTATTATTATCCC-TGTTTTACAAATAAGGAAATAAACT**CAGGGAGGTGAATGTGTT**CAAAGAT----ATATAGTTAAGAAAGTTAAAGAGACAGAATTCAAGCCTAAGTCTGTCTG-

MYCNAMP_NB2_241_Mmu ACATTCTTATTAAAATCACCTCAAACAGTGATGCTCATGTATACAAACTGAAT**TTGTGATAATAGTGTCTT**CTCCGAATGATAAATATTAAAGAAAGTATCATCTATAGTTTTACAG--TAAGGAGACTAGA

MYCNAMP_NB2_241_Rno CCATTCTTATTAAAAACACCTCAAACAGTGATGCTCATGTGAACAAACTGCCT**TAGTGATAGTACTGTCTT**CTCTGAATGATAAACATTAAAGCAAATAGCATC-ATAGTTTTACAG--TACG---ATTAAA

MYCNAMP_NB2_241_Hsa

ACAUUAUGAAGCAAGUAUUAUUAUCCCUGUUUUACAAAUAAGGAAAUAAACU**CAGGGAGGUGAAUGUGAU**CAAAGAUAGAUAUAUAGUUAAGAAAGUUAAAGAGACAGGAUUCAAGCCUAAGUCUGUCUG

......(((.(((..((((....((((((.((((............))))..))))))))))..)))..))).((((((((..............(((.....))).((.......))...)))))))). (-20.70)

MYCNAMP_NB2_241_Ptr

ACAUUAUGAAGCAAGUAUUAUUAUCCCUGUUUUACAAAUAAGGAAAUAAACU**CAGGGAGGUGAAUGUGUU**CAAAGAUAUAUAGUUAAGAAAGUUAAAGAGACAGAAUUCAAGCCUAAGUCUGUCUG

......((((.((....(((((.((((((.((((............))))..)))))))))))...))))))..........................(((((((.............))))))). (-19.52)

MYCNAMP_NB2_241_Mmu

ACAUUCUUAUUAAAAUCACCUCAAACAGUGAUGCUCAUGUAUACAAACUGAAU**UUGUGAUAAUAGUGUCUU**CUCCGAAUGAUAAAUAUUAAAGAAAGUAUCAUCUAUAGUUUUACAGUAAGGAGACUAGA

.......(((((..(((((......((((.((((....))))....)))).....)))))..)))))...(((((...((.((((.((((.(((........))).))))))))))....)))))..... (-22.10)

MYCNAMP_NB2_241_Rno

CCAUUCUUAUUAAAAACACCUCAAACAGUGAUGCUCAUGUGAACAAACUGCCU**UAGUGAUAGUACUGUCUU**CUCUGAAUGAUAAACAUUAAAGCAAAUAGCAUCAUAGUUUUACAGUACGAUUAAA

......................((((.((((((((..((....))...(((.((((((.........((......))........)))))).)))...)))))))).))))............... (-20.03)

MYCNAMP_NB2_241_Hsa --.--.--....----------(((.-(((..-((((..-.-----.---((-((((.--(--(--((-............))))..**)**-**)))**-**))**---**))))..)))**-**..)**)).((((((((..............(((.....))).----((.......)---

MYCNAMP_NB2_241_Ptr --.--.--....----------((((.((....((((-(-------.---((-((((.--(--(--((-............))))..**)-)))-)))**--**))))...))**-**)**-**)**))......--....--................----(((((((...........

MYCNAMP_NB2_241_Mmu --.--.--....----------.(((-((..(-((((..-.-----.---..-(((-(--.--(--((-(....--)----)))....))))-...**..)))))..))**-**)**-**))...(**(((--(...---((.(-(((-.---------((((.(((........))

MYCNAMP_NB2_241_Rno ......................((((.(((((-(((..((....))...(((.(**(((((.........((..**....))........)))))).)))...)))))))).)-)))----..--.--.----.-------.---------.----.....-..-----

MYCNAMP_NB2_241_Hsa ---).-..)-------))--))))-)-.-

MYCNAMP_NB2_241_Ptr ..)))))))------------------.-

MYCNAMP_NB2_241_Mmu ---).))))))))))....))))).....

MYCNAMP_NB2_241_Rno ------.----------------------

------------------------------------------------------------------------------------------------------------------------------------------------------------------------------------------------

---------

KELLY_276

---------

Identity: Ptr 0,97 Mml 0,88 Bta 0,68

TCGATTCCCACCCCTGACACCA

KELLY_276_Hsa CTTTTCACAGTGTTTTTTCAGTCATGAAAATGTAT-----TGGATTTCGTCAGTTATTTT-TCTGCATCTATTGAGATGA**TCGATTCCCACCCCTGACACCA**ACCTTGATTCTGTGAATATGGTGT-TTTCAGCGATTCATTTTCTTATTTTGAACCAAG

KELLY_276_Ptr CTTTTCACAGTGTTTTTTCAATCATGAAAATGTAT-----TGGACTTCATCAGTTATTTT-TCTGCATCTATTGAGATGA**TCGATTCCCACCCCTGACACCA**ACCTTGATTCTGTGAATATGGTGT-TTTCAGGGATTCATTTTCTTATTTTGAACCAAG

KELLY_276_Mml CTTTTCAGAGGATTTATTCAATCATGAAAACATAT-----TGGATTTTGCTAATTATTTT-TCTACATCTATTGAGATGA**TCAATTCCCACCC**-**TTACATCA**ACCTTGATTCTATAAATATGGTGT-TTTCAGCAATTCATTTTCTTATTTTGAACCAAG

KELLY_276_Bta ---------TTTTTTTTTTTATCATTAAAGTGTTTGGATTTGGATTTTGTCAGAAATTTTATCAGTGTATATTGAGATAA**TCGTGTTTCTTTCTTT**---**TGA**ATCTGTAG--GATTAATATAGTGTATTTTAGTGATTTGTTTTCTTGTACTGAATCATC

KELLY_276_Hsa

CUUUUCACAGUGUUUUUUCAGUCAUGAAAAUGUAUUGGAUUUCGUCAGUUAUUUUUCUGCAUCUAUUGAGAUGA**UCGAUUCCCACCCCUGACACCA**ACCUUGAUUCUGUGAAUAUGGUGUUUUCAGCGAUUCAUUUUCUUAUUUUGAACCAAG

...(((((((.(((..(((((((((....))).))))))....(((((.......((..(((((....)))))...)).........))))).........))).)))))))..((((.........((.......)).........)))).. (-28.96)

KELLY_276_Ptr

CUUUUCACAGUGUUUUUUCAAUCAUGAAAAUGUAUUGGACUUCAUCAGUUAUUUUUCUGCAUCUAUUGAGAUGA**UCGAUUCCCACCCCUGACACCA**ACCUUGAUUCUGUGAAUAUGGUGUUUUCAGGGAUUCAUUUUCUUAUUUUGAACCAAG

................(((((....(((((((....(((..((..(((........)))(((((....)))))...)).)))...((((((.....(((...((((...))))..)))....))))))...))))))).....)))))..... (-30.60)

KELLY_276_Mml

CUUUUCAGAGGAUUUAUUCAAUCAUGAAAACAUAUUGGAUUUUGCUAAUUAUUUUUCUACAUCUAUUGAGAUGA**UCAAUUCCCACCCUUACAUCA**ACCUUGAUUCUAUAAAUAUGGUGUUUUCAGCAAUUCAUUUUCUUAUUUUGAACCAAG

...(((((((.((((((..(((((.(((((...(((((......)))))...)))))..(((((....)))))..........................)))))..)))))).((.((....)).)).............)))))))..... (-20.20)

KELLY_276_Bta

UUUUUUUUUUUAUCAUUAAAGUGUUUGGAUUUGGAUUUUGUCAGAAAUUUUAUCAGUGUAUAUUGAGAUAA**UCGUGUUUCUUUCUUUUGA**AUCUGUAGGAUUAAUAUAGUGUAUUUUAGUGAUUUGUUUUCUUGUACUGAAUCAUC

.................................(((((.((((((((....((((.(((((((((...(((((.((.....(((....)))....)).)))))...)))))))...)).))))....)))).)).)).)))))... (-19.30)

KELLY_276_Hsa -------------.-..-----------------------------(((((((-.(((.-.--------((--(-(--------------((((--(....))).)))--))-)....(((((--.......((..-(((((....))))).**..)).........**

KELLY_276_Ptr -------------.-..----------------.............(((((....((((((--(....(((..((..(((........)))(((((....))))-).**..)).)))...(((((**--**(.....**(((..-.((((...)-)))-..-)))....))--

KELLY_276_Mml -------------.-..----------------(((((((-.----((((((.-.(((((.--(-(--(((--.-..-------------(((((......)))-)).--..))))-)-.-.---------------(((((....)))))..**............**

KELLY_276_Bta .................................(((((.(((----(((((....((((.(((------------(------------------------------------------(((**((...(((((.((.....(((....))**).-...)).)))))...

KELLY_276_Hsa **)))))....**.....))).)))--))))-..((((.........((.......)).-.....-...)))-)---------.-.

KELLY_276_Ptr -))))...--))))))).....)))))---------------------------------------..-.---------.-.

KELLY_276_Mml **........**....)))))..))--))))--.---((------.-((..--..)-).)).............))))))).....

KELLY_276_Bta )))))))...)).))))....)-))).)-)-------------------------------------.-)).)))))--...

------------------------------------------------------------------------------------------------------------------------------------------------------------------------------------------------

--------------

MYCNSC_NB2_148

--------------

Identity: Ptr 0,99 Mml 0,99 Mdo 0,91 Mmu 0,93 Rno 0,92 Cfa 0,94 Bta 0,96

ACCCAGCACCCCAGGTTTCCACAG

MYCNSC_NB2_148_Hsa TTGGTGGGGGAACTGGTTCTGGGTTCACCTCCCTGCTCATGGAACGTCTCTCAGTTGATTATGGCAAGAAGTCCAAGCTGGAGTTCTCCATTT**ACCCAGCACCCCAGGTTTCCACAG**CTGTAGTTGAGCCCTACAACTCCATCCTCACCACCCACACCACCCTGGAGCACTCTGATTGTGC

MYCNSC_NB2_148_Ptr TTGGTGGGGGAACTGGTTCTGGGTTCACCTCCCTGCTCATGGAACGTCTCTCAGTTGATTATGGCAAGAAGTCCAAGCTGGAGTTCTCCATTT**ACCCGGCGCCCCAGGTTTCCACAG**CTGTAGTTGAGCCCTACAACTCCATCCTCACCACCCACACCACCCTGGAGCACTCTGATTGTGC

MYCNSC_NB2_148_Mml TTGGTGGGGGAACTGGTTCTGGGTTCACCTCCCTGCTCATGGAACGTCTCTCAGTTGATTATGGCAAGAAGTCCAAGCTGGAGTTCTCCATTT**ACCCAGCGCCCCAGGTTTCCACAG**CTGTAGTTGAGCCCTACAACTCCATCCTCACCACCCACACCACCCTGGAGCACTCTGATTGTGC

MYCNSC_NB2_148_Mdo TTGGTGGTGGGACTGGCTCTGGTTTCACCTCTCTGCTGATGGAAAGGCTGTCTGTTGATTATGGCAAGAAGTCCAAGCTGGAGTTCTCCATTT**ACCCAGCCCCACAGGTTTCCACTG**CTGTGGTTGAGCCCTACAACTCCATTCTGACCACTCACACCACCCTGGAGCACTCTGATTGTGC

MYCNSC_NB2_148_Mmu TTGGTGGGGGAACTGGCTCTGGCTTCACCTCCCTGCTGATGGAGCGGCTCTCTGTGGATTACGGAAAGAAGTCCAAGCTGGAGTTCTCCATTT**ACCCAGCCCCCCAGGTTTCCACTG**CTGTGGTTGAGCCCTACAATTCCATCCTCACCACCCACACCACCCTGGAGCACTCTGATTGTGC

MYCNSC_NB2_148_Rno TTGGTGGGGGAACTGGCTCTGGGTTCACCTCCCTGCTGATGGAGAGGCTCTCTGTCGACTACGGAAAGAAGTCCAAGCTGGAGTTCTCCATTT**ACCCAGCCCCCCAGGTTTCCACTG**CTGTGGTTGAGCCCTACAATTCCATCCTCACCACCCACACCACCCTGGAGCACTCTGATTGTGC

MYCNSC_NB2_148_Cfa TTGGCGGGGGAACTGGTTCTGGGTTCACCTCCCTGCTGATGGAACGTCTCTCTGTCGATTATGGCAAGAAGTCCAAGCTAGAGTTCTCCATCT**ACCCTGCCCCCCAGGTGTCCACAG**CTGTAGTAGAGCCCTACAACTCCATCCTCACCACCCACACCACCCTGGAGCACTCTGATTGTGC

MYCNSC_NB2_148_Bta TTGGTGGGGGAACTGGTTCTGGGTTCACCTCCCTGCTGATGGAACGCCTCTCTGTCGATTATGGCAAGAAGTCCAAGCTGGAGTTCTCCATTT**ACCCAGCCCCCCAGGTTTCCACAG**CTGTCGTTGAGCCCTACAACTCTATCCTCACCACCCACACCACCCTGGAGCACTCTGATTGTGC

MYCNSC_NB2_148_Hsa

UUGGUGGGGGAACUGGUUCUGGGUUCACCUCCCUGCUCAUGGAACGUCUCUCAGUUGAUUAUGGCAAGAAGUCCAAGCUGGAGUUCUCCAUUU**ACCCAGCACCCCAGGUUUCCACAG**CUGUAGUUGAGCCCUACAACUCCAUCCUCACCACCCACACCACCCUGGAGCACUCUGAUUGUGC

.(((((((((....((((..(((((((.((....(((..(((((.........((((...((((..((((.(((.....))).)))))))).....)))).........))))).)))...)).)))))))...))))...)))))))))......((.....)).((((.......)))) (-53.97)

MYCNSC_NB2_148_Ptr

UUGGUGGGGGAACUGGUUCUGGGUUCACCUCCCUGCUCAUGGAACGUCUCUCAGUUGAUUAUGGCAAGAAGUCCAAGCUGGAGUUCUCCAUUU**ACCCGGCGCCCCAGGUUUCCACAG**CUGUAGUUGAGCCCUACAACUCCAUCCUCACCACCCACACCACCCUGGAGCACUCUGAUUGUGC

.(((((((((....((((..(((((((..(((........)))......((((((((...((((..((((.(((.....))).))))))))..(((.((....)).))).....)))))).)).)))))))...))))...)))))))))......((.....)).((((.......)))) (-54.70)

MYCNSC_NB2_148_Mml

UUGGUGGGGGAACUGGUUCUGGGUUCACCUCCCUGCUCAUGGAACGUCUCUCAGUUGAUUAUGGCAAGAAGUCCAAGCUGGAGUUCUCCAUUU**ACCCAGCGCCCCAGGUUUCCACAG**CUGUAGUUGAGCCCUACAACUCCAUCCUCACCACCCACACCACCCUGGAGCACUCUGAUUGUGC

.(((((((((....((((..(((((((.((....(((..(((((.........((((...((((..((((.(((.....))).)))))))).....)))).........))))).)))...)).)))))))...))))...)))))))))......((.....)).((((.......)))) (-55.07)

MYCNSC_NB2_148_Mdo

UUGGUGGUGGGACUGGCUCUGGUUUCACCUCUCUGCUGAUGGAAAGGCUGUCUGUUGAUUAUGGCAAGAAGUCCAAGCUGGAGUUCUCCAUUU**ACCCAGCCCCACAGGUUUCCACUG**CUGUGGUUGAGCCCUACAACUCCAUUCUGACCACUCACACCACCCUGGAGCACUCUGAUUGUGC

..(((((((.((.(((.((.((.....))........((((((..(((((...((.....((((..((((.(((.....))).))))))))..)).))))).....(((((((((....))))..))))).......))))))..))))).)).))))))).....((((.......)))) (-57.70)

MYCNSC_NB2_148_Mmu

UUGGUGGGGGAACUGGCUCUGGCUUCACCUCCCUGCUGAUGGAGCGGCUCUCUGUGGAUUACGGAAAGAAGUCCAAGCUGGAGUUCUCCAUUU**ACCCAGCCCCCCAGGUUUCCACUG**CUGUGGUUGAGCCCUACAAUUCCAUCCUCACCACCCACACCACCCUGGAGCACUCUGAUUGUGC

.(((((((((((.((.....(((((.((((....((((((((((.((((((...((((((.........))))))....)))))))))))).....)))).....))))..((((....))))..)))))...)).)))...))))))))......((.....)).((((.......)))) (-56.10)

MYCNSC_NB2_148_Rno

UUGGUGGGGGAACUGGCUCUGGGUUCACCUCCCUGCUGAUGGAGAGGCUCUCUGUCGACUACGGAAAGAAGUCCAAGCUGGAGUUCUCCAUUU**ACCCAGCCCCCCAGGUUUCCACUG**CUGUGGUUGAGCCCUACAAUUCCAUCCUCACCACCCACACCACCCUGGAGCACUCUGAUUGUGC

.(((((((((...(((....(((((((....((((..(((((((.....)))))))......(((......)))..(((((.((.........)))))))....))))...((((....)))).))))))).......))))))))))))......((.....)).((((.......)))) (-58.30)

MYCNSC_NB2_148_Cfa

UUGGCGGGGGAACUGGUUCUGGGUUCACCUCCCUGCUGAUGGAACGUCUCUCUGUCGAUUAUGGCAAGAAGUCCAAGCUAGAGUUCUCCAUCU**ACCCUGCCCCCCAGGUGUCCACAG**CUGUAGUAGAGCCCUACAACUCCAUCCUCACCACCCACACCACCCUGGAGCACUCUGAUUGUGC

.(((.(((((....((((..((((((..((....((((.((((.................((((..((((.((.......)).))))))))....((((.....))))..))))))))...))..))))))...))))...))))).)))......((.....)).((((.......)))) (-48.40)

MYCNSC_NB2_148_Bta

UUGGUGGGGGAACUGGUUCUGGGUUCACCUCCCUGCUGAUGGAACGCCUCUCUGUCGAUUAUGGCAAGAAGUCCAAGCUGGAGUUCUCCAUUU**ACCCAGCCCCCCAGGUUUCCACAG**CUGUCGUUGAGCCCUACAACUCUAUCCUCACCACCCACACCACCCUGGAGCACUCUGAUUGUGC

.(((((((((....((((..(((((((.......((((.(((((.((((..(((......((((..((((.(((.....))).)))))))).....)))......)))))))))))))......)))))))...))))...)))))))))......((.....)).((((.......)))) (-59.32)

MYCNSC_NB2_148_Hsa .(((((((((....((-((-..(((((((.((....-----------(---((..(((((.........(-(-((---...((((..-((((.(((.....))).))))--**)**--**)**-**)**)-..**..**------**.)**-**)))**---------**.**----**.**--**.**-**..**---------

MYCNSC_NB2_148_Ptr .(((((((((....((-((-..(((((((..(((........)))......(((-((((--(----------------...((((..-((((.(((.....))).))))--**)**--**)**-**)**)-..----------------------**(((.(**-**(**--**....**-----**))**--

MYCNSC_NB2_148_Mml .(((((((((....((-((-..(((((((.((....-----------(---((..(((((.........(-(-((---...((((..-((((.(((.....))).))))--**)**--**)**-**)**)-..**..**------**.)**-**)))**---------**.**----**.**--**.**-**..**---------

MYCNSC_NB2_148_Mdo ..(((((((----.((-.---((-(.((---.((..--...)-)........((-((((.-.----(((((...((.....((((..-((((.(((.....))).))))--**)**--**)**-**)**)-..**)**---------**).))))).....(((((**-**((((..**.-.))))..)

MYCNSC_NB2_148_Mmu .((((((((----(((.((.....((((---(-------------------.((-((....(-(-(((((-(-((---.((((((...((((((.........))))))....))-)))-)----------))**)))**)-------.----.--**.**-**..**---------

MYCNSC_NB2_148_Rno .(((((((((...(((-..-..(((((((...-------------------.---(((---(----------------..---------(((((((.....))))))).--.--....-(((......)))-.-.--------(((((.((...**...**...**)))**--

MYCNSC_NB2_148_Cfa .(((.(((((....((-((-..((((((..((....-----------(---(((.((((...........-.-..---...((((..-((((.((.......)).))))--**)**--**)**-**)**)-..**..**--------------------**(((**-**(**-**.**--**.**-**..**-----**.)**--

MYCNSC_NB2_148_Bta .(((((((((....((-((-..(((((((.......-----------(---(((.(((((.(-(-((..--(-((......((((..-((((.(((.....))).))))--**)**--**)**-**)**)-..**..**------**.)**-**)).**---------**.**----**.**--**.**-**..**---------

MYCNSC_NB2_148_Hsa **.**---**...)**---**)))).**-**))**)----..-.---)).)))))))...))--))...)))))))))......((.....)).((((.......))))

MYCNSC_NB2_148_Ptr **.))).....**-**)))**))).))-----.--------)-))))))...))--))...)))))))))......((.....)).((((.......))))

MYCNSC_NB2_148_Mml **.**---**...)**---**)))).**-**))**)----..-.---)).)))))))...))--))...)))))))))......((.....)).((((.......))))

MYCNSC_NB2_148_Mdo )))).......)))))-)------.--------.-))-)-)-)--.--))--.-)))))))-.-----------....((((.......))))

MYCNSC_NB2_148_Mmu **)**---**)))..**-**...)))**-**)..((((..**..))))..)))))-...)-).-)))...))))))))......((.....)).((((.......))))

MYCNSC_NB2_148_Rno **))))....**---**)))).**-**..**-**((((..**..))))-.))))))).......))--))))))))))......((.....)).((((.......))))

MYCNSC_NB2_148_Cfa **)**-**))**--**..**---**)))))**-**))**)----..-.---))..))))))...))--))...))))).)))......((.....)).((((.......))))

MYCNSC_NB2_148_Bta **)**---**))))**---**)))))-))**)----..-.---...)))))))...))--))...)))))))))......((.....)).((((.......))))

------------------------------------------------------------------------------------------------------------------------------------------------------------------------------------------------

--------------

MYCNSC_NB3_226

--------------

Identity: Ptr 1 Mml 0,97 Mmu 0,90 Rno 0,91 Cfa 0,93 Bta 0,92

CTGCCCTGGCCCGAGGGACCGA

MYCNSC_NB3_226_Hsa GACATGCT-CCATCGGATGAATTGTT--GGTGTTAGCCCTGCGGCCCCACGCACCAGGGTAAGAGAGACTCTCGCTTC**CTGCCCTGGCCCGAGGGACCGA**CTGGCTGGGCCTGCCTTCTGCCCAGCTCACCGGTCACAGAAGAGAGTTTGGACATGCATT-CTACTCTTGCCCGATGAA

MYCNSC_NB3_226_Ptr GACATGCT-CCATCGGATGAATTGTT--GGTGTTAGCCCTGCGGCCCCACGCACCAGGGTAAGAGAGACTCTCGCTTC**CTGCCCTGGCCCGAGGGACCGA**CTGGCTGGGCCTGCCTTCTGCCCAGCTCACCGGTCACAGAAGAGAGTTTGGACATGCATT-CTACTCTTGCCCGATGAA

MYCNSC_NB3_226_Mml GACATGCT-CCTTCGGATGAATTGCT--GGTGTTAGCCCTGCGGCCCCACGCACCAGGGTAAGAGAGAGTCTCGCTTC**CTGCCCTGGCCCGAGGGACCGA**CTGGCTGGGCCTGCCTCCTGCCCAGCTCACCGGTCACAGAAGAGAGTTTGGACATACATT-CTACTCTTGCCCGATGAA

MYCNSC_NB3_226_Mmu GACACACTTCCTTCGGATGAATTTTT-TGGTGTTAGCCCTGCGGCCCCACGCACCAGGGTAAGAGAGA--CTCACTTC**CTGCCCTGGCCCGAGGGACCGA**CTGGCTGGGCCTGCTTTCTGCCCAGCTCACCTGTCATGGAAGAGAGTTTGGACACACTCCACT---CTTGCCCCTTGAA

MYCNSC_NB3_226_Rno GACACACTTCCATTGGATGAATTTTTCTGGTGTTAGCCCTGCGGCCCCACGCACCAGGGTAAGAGAGA--CTCGCTTC**CTGCCCTGGCCCGAGGGACCGA**CTGGCTGGGCCTGCTTTCTGCCCAGCTTACCTGTCACGGAAGAGAGTCTGGACACACACGACTACTCTTGCCCCACGGA

MYCNSC_NB3_226_Cfa GACACACT-CCTTTGGATGAATT-----GGTGTTAGCCCTGCGGCCCCACGCACCAGGGTAAGAGAGACTCTCGCTTC**CTGCCCTGGCCCGAGGGACCGA**CTGGCTGGGCCTGCCTTCTGCCCAGCTCACTGGTCATGGAAGA--GTTCGGACACGCACT-CCACTCTTGCCTGATGAA

MYCNSC_NB3_226_Bta GACCTGCT-CCTTCGGATGAATTTTT--GGTGTTAGCCCTGCGGCCCCACGCACCAGGGTAAGAGAGA--CTCACTTC**CTGCCCTGGCCCGAGGGACCGA**CTGGCTGGGCCTGCCTTCTGCCCAGGTCACCGGTCGTGGAAGAGAGTTTGGACACACACT-CTACCTTTGCCTGATGAA

MYCNSC_NB3_226_Hsa

GACAUGCUCCAUCGGAUGAAUUGUUGGUGUUAGCCCUGCGGCCCCACGCACCAGGGUAAGAGAGACUCUCGCUUC**CUGCCCUGGCCCGAGGGACCGA**CUGGCUGGGCCUGCCUUCUGCCCAGCUCACCGGUCACAGAAGAGAGUUUGGACAUGCAUUCUACUCUUGCCCGAUGAA

.........((((((......((((((((.........(((.(((.((..((((((((((.(((...))).))...))))))))..)).))).)))...((((((((.........)))))))))))))).))...(((((((..((......))..)).)))))..)))))).. (-65.30)

MYCNSC_NB3_226_Ptr

GACAUGCUCCAUCGGAUGAAUUGUUGGUGUUAGCCCUGCGGCCCCACGCACCAGGGUAAGAGAGACUCUCGCUUC**CUGCCCUGGCCCGAGGGACCGA**CUGGCUGGGCCUGCCUUCUGCCCAGCUCACCGGUCACAGAAGAGAGUUUGGACAUGCAUUCUACUCUUGCCCGAUGAA

.........((((((......((((((((.........(((.(((.((..((((((((((.(((...))).))...))))))))..)).))).)))...((((((((.........)))))))))))))).))...(((((((..((......))..)).)))))..)))))).. (-65.30)

MYCNSC_NB3_226_Mml

GACAUGCUCCUUCGGAUGAAUUGCUGGUGUUAGCCCUGCGGCCCCACGCACCAGGGUAAGAGAGAGUCUCGCUUC**CUGCCCUGGCCCGAGGGACCGA**CUGGCUGGGCCUGCCUCCUGCCCAGCUCACCGGUCACAGAAGAGAGUUUGGACAUACAUUCUACUCUUGCCCGAUGAA

.......((..((((.......(((((((.........(((.(((.((..((((((((((.(((...))).))...))))))))..)).))).)))...((((((((.........))))))))))))))).....(((((((..((......))..)).)))))..)))).)). (-65.30)

MYCNSC_NB3_226_Mmu

GACACACUUCCUUCGGAUGAAUUUUUUGGUGUUAGCCCUGCGGCCCCACGCACCAGGGUAAGAGAGACUCACUUC**CUGCCCUGGCCCGAGGGACCGA**CUGGCUGGGCCUGCUUUCUGCCCAGCUCACCUGUCAUGGAAGAGAGUUUGGACACACUCCACUCUUGCCCCUUGAA

((((....((((((((............((((..(((....)))...)))).(((((((.((..((.....))..))))))))).))))))))......((((((((.........))))))))....))))..((..((((((..(((.....)))))))))..))...... (-63.10)

MYCNSC_NB3_226_Rno

GACACACUUCCAUUGGAUGAAUUUUUCUGGUGUUAGCCCUGCGGCCCCACGCACCAGGGUAAGAGAGACUCGCUUC**CUGCCCUGGCCCGAGGGACCGA**CUGGCUGGGCCUGCUUUCUGCCCAGCUUACCUGUCACGGAAGAGAGUCUGGACACACACGACUACUCUUGCCCCACGGA

((((..((..(((..((........))..)))..)).....(((.(((.((..(((((((.((..((.....))..)))))))))..)).))).)))...((((((((.........))))))))....))))..(((((((((((((......)).)))).))))).))....... (-67.40)

MYCNSC_NB3_226_Cfa

GACACACUCCUUUGGAUGAAUUGGUGUUAGCCCUGCGGCCCCACGCACCAGGGUAAGAGAGACUCUCGCUUC**CUGCCCUGGCCCGAGGGACCGA**CUGGCUGGGCCUGCCUUCUGCCCAGCUCACUGGUCAUGGAAGAGUUCGGACACGCACUCCACUCUUGCCUGAUGAA

.......((((((((........((((..(((....)))...)))).((((((((((.(((...))).))...)))))))).))))))))..((((((((((((.........))))))))....))))..((((((((..(((.......))))))))).))....... (-60.30)

MYCNSC_NB3_226_Bta

GACCUGCUCCUUCGGAUGAAUUUUUGGUGUUAGCCCUGCGGCCCCACGCACCAGGGUAAGAGAGACUCACUUC**CUGCCCUGGCCCGAGGGACCGA**CUGGCUGGGCCUGCCUUCUGCCCAGGUCACCGGUCGUGGAAGAGAGUUUGGACACACACUCUACCUUUGCCUGAUGAA

((((((.((((((((...........((((..(((....)))...)))).(((((((.((..((.....))..))))))))).))))))))......(((.(((....)))...)))))))))...(((...((...(((((.((....)).))))).))...)))....... (-63.50)

MYCNSC_NB3_226_Hsa .........((((((......(((((((-------------------------(.........(((.(((.-----(----(----------------------..(((((((-(((.(((...))).))..**.))))))))..)).))).)))**----**.**..(((((

MYCNSC_NB3_226_Ptr .........((((((......(((((((-------------------------(.........(((.(((.-----(----(----------------------..(((((((-(((.(((...))).))..**.))))))))..)).))).)))**----**.**..(((((

MYCNSC_NB3_226_Mml .......((..((((.......((((((-------------------------(.........(((.(((.-----(----(----------------------..(((((((-(((.(((...))).))..**.))))))))..)).))).)))**----**.**..(((((

MYCNSC_NB3_226_Mmu -----------------------((((.----------------------------.-..-(-(((((((............((((..(((....)))...)))).(((((((.((..((.....))..**))**---**))))))).)**----**)))))))....**..(((((

MYCNSC_NB3_226_Rno ----------------------((((..((..(((..((........))..)))..)).....(((.(((.-----(----(----------------------..(((((((.((..((.....))..**))**---**)))))))..)).))).)))**----**.**..(((((

MYCNSC_NB3_226_Cfa -----------------------.....----------------------------.-.--(-(((((((.......----.((((..(((....)))...)))).(((((((-(((.(((...))).))..**.)))))))).)**----**)))))))..((**(((((((

MYCNSC_NB3_226_Bta -----------------------(((((----------------------------(-.--(-(((((((..........-.((((..(((....)))...)))).(((((((.((..((.....))..**))**---**))))))).)**----**)))))))....**..(((.-

MYCNSC_NB3_226_Hsa (((.........)))-))))))-------)))-)).))...(((-((---((.--.(-(...-...))-.-.)).)))))..))))))..

MYCNSC_NB3_226_Ptr (((.........)))-))))))-------)))-)).))...(((-((---((.--.(-(...-...))-.-.)).)))))..))))))..

MYCNSC_NB3_226_Mml (((.........)))-))))))-------)))-))).....(((-((---((.--.(-(...-...))-.-.)).)))))..)))).)).

MYCNSC_NB3_226_Mmu (((.........)))-)))))...--.)---)-))-..-((..(-((---(((..((-(...--..))-)-)))-)))..-))..-....

MYCNSC_NB3_226_Rno (((.........)))-))))).-------...)))-)..(((((-((---(((--((-(...-...))-.)))).))))).)).......

MYCNSC_NB3_226_Cfa (((.........)))-)))))....))))-------..-((--(-((---(((..((-(.......))-)-)))-))).)--).......

MYCNSC_NB3_226_Bta ((---(....)--))...))))-------)))-))-...(((...((...(((--((.((..-..)).))-))).))...))).......

------------------------------------------------------------------------------------------------------------------------------------------------------------------------------------------------

-------------

MYCNSC_NB5_41

-------------

Identity: Ptr 1 Mml 0,99 Mmu 0,46 Rno 0,46 Cfa 0,58

TCACCCCATAAACACCA

MYCNSC_NB5_41_Hsa GGGAGCTCAGAAGCTTCACCTAAGAAT-TAGGGTTCATC----AAT-GATT**TCACCCCATAAACACCA**ATTC---TTCTATCTTGTTCAGGAAATG--GTGATTATGGTGCAAAACAAACCACCTGAA

MYCNSC_NB5_41_Ptr GGGAGCTCAGAAGCTTCACCTAAGAAT-TAGGGTTCATC----AAT-GATT**TCACCCCATAAACACCA**ATTC---TTCTATCTTGTTCAGGAAATG--GTGATTATGGTGCAAAACAAACCACCTGAA

MYCNSC_NB5_41_Mml GGGAGCTCAGAAGCTTCACCTAAGAAT-TAGGGTTCATT----AAT-GATT**TCACCCCATAAACACCA**ATTC---TTCTATCTTGTTCAGGAAATG--GTGATTATGGTGCAAAACAAACCACCTGAA

MYCNSC_NB5_41_Mmu AAAGTGTCACCATTTCATCCTTCAAAG-CCGCCTTTTTTGGGAAATTGAGA**TTATGGCGCAAACACCA**CCTGAGATTTTAATGTCATCTGCTACAC--TTTTCTTTCTTGCTTACCAG----------

MYCNSC_NB5_41_Rno AAAATGTCATCATTTCACCCTTCAAAG-CTGCCTTTTCTGG-AAATCGACC**TTCCGGTGCAAACACCA**CTTGAGATTTTAATGTCATATGCTGCAC--GTTGTTTTCTTGCTTACCAG----------

MYCNSC_NB5_41_Cfa ---------AAATGGCAAGGAAAGCAGGCTGGGTTTAGGAAATGTGGGGTT**TTGTCTCAGCTCTACCA**TTAA--CTTGTGTTTTAGTCAGCTATTGCCTTAATTATGCTGCAAAACAAACCACCCAAA

MYCNSC_NB5_41_Hsa

GGGAGCUCAGAAGCUUCACCUAAGAAUUAGGGUUCAUCAAUGAUU**UCACCCCAUAAACACCA**AUUCUUCUAUCUUGUUCAGGAAAUGGUGAUUAUGGUGCAAAACAAACCACCUGAA

.((((((....)))))).........(((((..(((....))).......((((((.(((((.(((((...........))))).))))).))))))..............))))). (-27.70)

MYCNSC_NB5_41_Ptr

GGGAGCUCAGAAGCUUCACCUAAGAAUUAGGGUUCAUCAAUGAUU**UCACCCCAUAAACACCA**AUUCUUCUAUCUUGUUCAGGAAAUGGUGAUUAUGGUGCAAAACAAACCACCUGAA

.((((((....)))))).........(((((..(((....))).......((((((.(((((.(((((...........))))).))))).))))))..............))))). (-27.70)

MYCNSC_NB5_41_Mml

GGGAGCUCAGAAGCUUCACCUAAGAAUUAGGGUUCAUUAAUGAUU**UCACCCCAUAAACACCA**AUUCUUCUAUCUUGUUCAGGAAAUGGUGAUUAUGGUGCAAAACAAACCACCUGAA

.((((((....)))))).........(((((..(((....))).......((((((.(((((.(((((...........))))).))))).))))))..............))))). (-27.70)

MYCNSC_NB5_41_Mmu

AAAGUGUCACCAUUUCAUCCUUCAAAGCCGCCUUUUUUGGGAAAUUGAGA**UUAUGGCGCAAACACCA**CCUGAGAUUUUAAUGUCAUCUGCUACACUUUUCUUUCUUGCUUACCAG

...((((((..((((((((((..((((....))))...))))...))))))..)))))).........(((.(((......)))....((................))....))) (-18.29)

MYCNSC_NB5_41_Rno

AAAAUGUCAUCAUUUCACCCUUCAAAGCUGCCUUUUCUGGAAAUCGACC**UUCCGGUGCAAACACCA**CUUGAGAUUUUAAUGUCAUAUGCUGCACGUUGUUUUCUUGCUUACCAG

.(((((....))))).....................((((.............((((....))))....((((...((((((((.....)).))))))...)))).....)))) (-17.00)

MYCNSC_NB5_41_Cfa

AAAUGGCAAGGAAAGCAGGCUGGGUUUAGGAAAUGUGGGGUU**UUGUCUCAGCUCUACCA**UUAACUUGUGUUUUAGUCAGCUAUUGCCUUAAUUAUGCUGCAAAACAAACCACCCAAA

....(((((....(((.(((((((...((..(((((((((((.......))))))).))))..)).....))))))).))).))))).............................. (-25.00)

MYCNSC_NB5_41_Hsa .((((((....))))))...-.--..-...------(((-((..-(((....)))...-**.**----**...((((((.(((((**.(((((...........))))).))))).))-))))..............))-)))-----------------------------------------------------.-

MYCNSC_NB5_41_Ptr .((((((....))))))...-.--..-...------(((-((..-(((....)))...-**.**----**...((((((.(((((.**(((((...........))))).))))).))-))))..............))-)))-----------------------------------------------------.-

MYCNSC_NB5_41_Mml .((((((....))))))...-.--..-...------(((-((..-(((....)))...-**.**----**...((((((.(((((.**(((((...........))))).))))).))-))))..............))-)))-----------------------------------------------------.-

MYCNSC_NB5_41_Mmu ---.----.-.--------------------------((-((((.-.-----------------(--((((--(((((-..((((.-.-.-.-)---)))...))))...)))))--**)**-**..)))**------**)**-**))........**.(((.(((......)))....((................))....)))

MYCNSC_NB5_41_Rno .-(((((....))))).....................((-((.........**....((((....)))).**...((-((...(((((((-(.....)).))))))...))-))------.....-)-------)-))--------------------------------------------------------

MYCNSC_NB5_41_Cfa ---.----.-.----.-(((-(--(.-...------(((.(((((-(-----------------(--...((..((((-(((((((-**.......))**-**))))).))**))..)-)...-..))))))------).))).))))).--.-.-...--.-..----............-.---..--....--..

------------------------------------------------------------------------------------------------------------------------------------------------------------------------------------------------

-------------

MYCNSC_NB5_64

-------------

identity: Ptr 0,92 Mdo 0,42 Cfa 0,85 Bta 0,85

AAGCTAATTTTTTGAGGCC

MYCNSC_NB5_64_Hsa --------------------------------------------------CTTTCGCCTTTTACTAAAGATTTCCGTGGAGAGGAACAACTCTGAGTCTT**AAGCTAATTTTTTGAGGCC**TTGTTCCGACAAGGCTATATAAAGCTGTTAAAAAATCAGATTGACTTCATTTAGGGTGTT--

MYCNSC_NB5_64_Ptr --------------------------------------------------CTTTCGCCTTTTACTAAAGATTTCCGTGGAGAGGAACAACTCTGAGTCTT**AAGCCAATTTTTTGAGGCC**TTGCTCCGGCAAGGCTACACAAAGCTGTTGAAAAATTAGATTCAGTTCGTTTAGG-TGTT--

MYCNSC_NB5_64_Mdo --------------------------------------GTTCTCTGTTAGAAAAACATTTATAGTAGCAACTTTTGTAATGGAGAAGAACTG-GAAACTA**AAGG**-**AGTGTTCATTAACT**GAGGAAGGACTAAGCAAGTTTTATTTATATATTAAAAAGTATCAGAGACACTTGGAAAGA--

MYCNSC_NB5_64_Cfa ---------------------------------------------------TTTCGCCTTTTACTAAAGATTTCCGTGGAGAGGAACAACTCTGAGTCTTAAACC**AATTTTTTGAGGCC**TTGTGTCAGCAAGGCTACGTGATGTCTCAAAAAAAAGACAATTATAAGCTGTTTTGAAATGT

MYCNSC_NB5_64_Bta AATTACTAGGGGGTAGGTTATACTCTGGTTTCTCTTCAGATCGTATAAATCTTTCGCCTTTTACTAAAGATTTCCGTGGAGAGGAACAACTCTGAGTCTAAAACC**AATTTTTTGAGGCT**TTGCTTCGGCAAAGCTAC-TTATCTTGGTTAAAAATAAGA----------------------

MYCNSC_NB5_64_Hsa

CUUUCGCCUUUUACUAAAGAUUUCCGUGGAGAGGAACAACUCUGAGUCUU**AAGCUAAUUUUUUGAGGCC**UUGUUCCGACAAGGCUAUAUAAAGCUGUUAAAAAAUCAGAUUGACUUCAUUUAGGGUGUU

....((((((...................((((......))))(((((.....((.(((((((((((((((((....))))))))...........))))))))).))...))))).....)))))).. (-28.70)

MYCNSC_NB5_64_Ptr

CUUUCGCCUUUUACUAAAGAUUUCCGUGGAGAGGAACAACUCUGAGUCUU**AAGCCAAUUUUUUGAGGCC**UUGCUCCGGCAAGGCUACACAAAGCUGUUGAAAAAUUAGAUUCAGUUCGUUUAGGUGUU

....(((((...((.......((((.......)))).....((((((((.....((((.(((((.((((((((....))))))))...)))))..)))).......))))))))...))..))))).. (-36.20)

MYCNSC_NB5_64_Mdo

GUUCUCUGUUAGAAAAACAUUUAUAGUAGCAACUUUUGUAAUGGAGAAGAACUGGAAACUA**AAGGAGUGUUCAUUAACU**GAGGAAGGACUAAGCAAGUUUUAUUUAUAUAUUAAAAAGUAUCAGAGACACUUGGAAAGA

.((((((((((.((((.................)))).))))))))))................(((((((......((((...((((((.....)))))).((((.....)))).....)))).)))))))....... (-20.23)

MYCNSC_NB5_64_Cfa

UUUCGCCUUUUACUAAAGAUUUCCGUGGAGAGGAACAACUCUGAGUCUUAAACC**AAUUUUUUGAGGCC**UUGUGUCAGCAAGGCUACGUGAUGUCUCAAAAAAAAGACAAUUAUAAGCUGUUUUGAAAUGU

..................(((((.....((((......))))..(((((.......((((((((((((((((....)))))))..........))))))))))))))................))))).. (-25.91)

MYCNSC_NB5_64_Bta

AAUUACUAGGGGGUAGGUUAUACUCUGGUUUCUCUUCAGAUCGUAUAAAUCUUUCGCCUUUUACUAAAGAUUUCCGUGGAGAGGAACAACUCUGAGUCUAAAACC**AAUUUUUUGAGGCU**UUGCUUCGGCAAAGCUACUUAUCUUGGUUAAAAAUAAGA

....((((((.(((...(((.((((..(((((((((((...((...((((((((...........)))))))).)))))))))))))......)))).))).)))..........((((((((....))))))))......))))))........... (-43.40)

MYCNSC_NB5_64_Hsa ---.---...--------((((((...-----------------................--(((--(.---.--..-.---.-)-)))--------------------------------(((**((**..**...((.((((**-**((((**-**(((((**((((....))))))))

MYCNSC_NB5_64_Ptr ---.---...--------(((((...(-----------------(.......----------(((--(.---.--..-..--.-)-)))-------------.....--------------(((((**((**(.**....((((.(((((.((((**((((....))))))))

MYCNSC_NB5_64_Mdo -------------------.------------------------((((((((-----((.--((((.......-.......-...)))).))--))))))))......-...**..**.-.**..**-**.(((((((......((**(--(...---((((((.....)-))))).

MYCNSC_NB5_64_Cfa ..................(((((....----------------------.------------(((--(.---.--..-.---.-)-)))-------------.------------------.(**((**((.....**..((((**-**((((**--**((((**((((....))))))).

MYCNSC_NB5_64_Bta ---.---..----------.((((((.(((...(((.((((..(((((((((((...((...((((((((...........)))))))).)))))))))))))......)))**).**))).)))**........**-**.**--**.**--**(**----**(**----**((((**((..-..)-)))))-

MYCNSC_NB5_64_Hsa ..-.........))))))--))).)).-..-)--)))--)...----.-.)--))))).---.----

MYCNSC_NB5_64_Ptr ..-.-----)---))))..)))).......)))))))--)...--)-)-..--))))).---.----

MYCNSC_NB5_64_Mdo ((((.....)))).....)-)))---.----))))))--)-------------......---.----

MYCNSC_NB5_64_Cfa ..-.......)--)))))--)))---)------))))................))))).---.----

MYCNSC_NB5_64_Bta -)------------------)..---.-------...--)---------))--)))...........

------------------------------------------------------------------------------------------------------------------------------------------------------------------------------------------------

--------------

MYCNAMP_NB4_70

--------------

Identity: Ptr 1 Mml 0,97 Mmu 0,79 Rno 0,76 Cfa 0,78

CCTGTGTTTTGTTGGTAGCCTGTGTTAC

MYCNAMP_NB4_70_Hsa ----------------------------AGTTTTAATCATGCAGTTAATAAAATATTTATTAAGTAATCCTGTTTTGTTGGTAG**CCTGTGTTTTGTTGGTAGCCTGTGTTAC**TCCCAAAGGTCATAACCTTCAATAATATAGTTAATAAAATG

MYCNAMP_NB4_70_Ptr TAAGATGTATTCGCTTGGTAGCTAATCTAGTTTTAATCATGCAGTTAATAAAATATTTATTAAGTAATCCTGTTTTGTTGGTAG**CCTGTGTT**------------------**AC**TCCCAAAGGTCATAACCTTCAATAATATAGTTAATAAAATG

MYCNAMP_NB4_70_Mml TAAGATGTATTCTTTTGGTAGCTAATCTAGTTTTAATCATGCAGTTAATAAAATATTTATTAACTAATCCTGTTTTGTTGGTAG**CCTGTATT**------------------**AC**TCCCAAAGGTC----------ATAATTTAGTTAATAAAATG

MYCNAMP_NB4_70_Mmu TAAGTTGTATTATCTTGGTAGACAATCTAGTTTTAATTATGCAGCTAATAAAATATTTATTAAGTAATTCAGTGTACCTGATAC**CCAGTGTT**------------------**AC**ATTCAATGGTCATGACCTTTAAGAATTTAGCTAATAAATA-

MYCNAMP_NB4_70_Rno TAAGTTGTATTCTCCTGGTAGATAATCTAGTTTTAATTATGCAACTAATAAAATATTTACTAAGTAATTCAGAGTTCCTGATAC**TCAGTGTT**------------------**AC**TCTCAAAGGTCATGACCTTTAAGAATTTAGCTGAAGTG---

MYCNAMP_NB4_70_Cfa TAAGATGTATTCTCTTGGTAGCTAATCTATTTTCAATCATATAGCTAATGAAATAT-----------TTCAGACTTCCTGGTAG**CATGTATT**------------------**AC**TCCCAAAGGTCATGACCTCCAAAAATTTAGTGAGAAAAATG

MYCNAMP_NB4_70_Hsa

AGUUUUAAUCAUGCAGUUAAUAAAAUAUUUAUUAAGUAAUCCUGUUUUGUUGGUAG**CCUGUGUUUUGUUGGUAGCCUGUGUUAC**UCCCAAAGGUCAUAACCUUCAAUAAUAUAGUUAAUAAAAUG

.((((((....(((..((((((((...)))))))))))...((((.(((((((.((..(((((((((..((((((....))))))..)))))..))))..))))))))).))))....)))))). (-22.40)

MYCNAMP_NB4_70_Ptr

UAAGAUGUAUUCGCUUGGUAGCUAAUCUAGUUUUAAUCAUGCAGUUAAUAAAAUAUUUAUUAAGUAAUCCUGUUUUGUUGGUAG**CCUGUGUUAC**UCCCAAAGGUCAUAACCUUCAAUAAUAUAGUUAAUAAAAUG

...(((......((.(((((((((...)))))...)))).))..((((((((...))))))))...)))..((((((((((....((((((((......(((((....)))))...)))))))))))))))))). (-19.50)

MYCNAMP_NB4_70_Mml

UAAGAUGUAUUCUUUUGGUAGCUAAUCUAGUUUUAAUCAUGCAGUUAAUAAAAUAUUUAUUAACUAAUCCUGUUUUGUUGGUAG**CCUGUAUUAC**UCCCAAAGGUCAUAAUUUAGUUAAUAAAAUG

.....(((((....((((.(((((...)))))))))..)))))............((((((((((((...(((((((..(((((......)))))..)))))..))....))))))))))))... (-18.40)

MYCNAMP_NB4_70_Mmu

UAAGUUGUAUUAUCUUGGUAGACAAUCUAGUUUUAAUUAUGCAGCUAAUAAAAUAUUUAUUAAGUAAUUCAGUGUACCUGAUAC**CCAGUGUUAC**AUUCAAUGGUCAUGACCUUUAAGAAUUUAGCUAAUAAAUA

..((((((((........(((.....))).........)))))))).......(((((((((.(((((((.(((((((((.....))).)).)))).....(((....))).....)))))..))))))))))) (-23.13)

MYCNAMP_NB4_70_Rno

UAAGUUGUAUUCUCCUGGUAGAUAAUCUAGUUUUAAUUAUGCAACUAAUAAAAUAUUUACUAAGUAAUUCAGAGUU**CCUGAUAC**UCAGUGUUACUCUCAAAGGUCAUGACCUUUAAGAAUUUAGCUGAAGUG

..((((((((....((((........))))........))))))))......................((((.....)))).(((((((.....(((.((((((....)))))).))).....)))).))). (-26.40)

MYCNAMP_NB4_70_Cfa

UAAGAUGUAUUCUCUUGGUAGCUAAUCUAUUUUCAAUCAUAUAGCUAAUGAAAUAUUUCAGACUU**CCUGGUAGC**AUGUAUUACUCCCAAAGGUCAUGACCUCCAAAAAUUUAGUGAGAAAAAUG

.........(((((....((((((.................))))))..........((((((((..(((.((.........)).))).))))).)))................)))))..... (-17.13)

MYCNAMP_NB4_70_Hsa -.----((-(((---(..-.--.(((..((((-((-(-(...)-))-)))---)))--))-------------.-------.----.------------------((((.((-(((((.((**..(((((((((-..--((((((....)))))**-)..)))--))-.

MYCNAMP_NB4_70_Ptr -------.-..--(((......((.((((--(-((-((-...-)))-))..--.)))).))-.-.((((((((...))))))))...)))-.-------------.(((-((-((((-(....**(**-**(((((((**......(((((....)))))-...)))--))--

MYCNAMP_NB4_70_Mml -------.-...---.((-(--((....((((.((-(((...))))-)))---)).--.))))).-----..-.-----.-.----..---.-----------...(((-((-((((-(((...-(((((((-..--(((((**......))))**-)..)))--))-.

MYCNAMP_NB4_70_Mmu -------.-.-----(((-(--((((........(-((.....))).....--....))))))))-----..-.-----.-.----..---(-----------((((((-((.((((-((--(.-----(((-(---(((((.-...**.))).)).))**)).....(

MYCNAMP_NB4_70_Rno -------.-.-----(((-(--((((....-(-(((........))-))........))))))))--......................-((((....**.))))**--**.((**(--(-(((.-...-.----(((.(------(((((....)))))----).)--))--

MYCNAMP_NB4_70_Cfa .........((((--(..-.--.((-((((..-..-..........-...---))))-))-------------.-------.----.------------------....--.--..--((--((((((**..((**-**(**---**.((.**..-......))--.))).---)--

MYCNAMP_NB4_70_Hsa -.------))))..))))))--))).))))....)))))--).

MYCNAMP_NB4_70_Ptr -)-------))----)-)))--)))-)))-------------.

MYCNAMP_NB4_70_Mml -.------))....))))))--)))-)))---------.--..

MYCNAMP_NB4_70_Mmu ((....))).....)))))..))))-)))))))----------

MYCNAMP_NB4_70_Rno -.-------..----.-.))--)).-)))-------------.

MYCNAMP_NB4_70_Cfa -)-------))).)-))................))))).....

------------------------------------------------------------------------------------------------------------------------------------------------------------------------------------------------

--------------

MYCNAMP_NB2_61

--------------

Identity: Ptr 0,98 Mml 0,96

TCAAAACTGAGGGGCATTTTCT

MYCNAMP_NB2_61_Hsa TAACCACGTAGGAAGAGTTTGAAGTCAGACATGACATTCAGACTGAGGTCC**TCAAAACTGAGGGGCATTTTCT**GTGGTTTGAAAGGAAAGTGCACCCAGTTTTGGGGATGTCAATTGTGAATCCTCA

MYCNAMP_NB2_61_Ptr TAACCACGTAGGAAGAGTTTGAAGTCAGACGTGACATTCAGACTGAGGTCC**TCAAAACTGAGGGGCATTTTCT**GTGGTTTGAAAGGAAAGTGCACCCAGTTTTGGGGATGTCAATTGTGAATACTCA

MYCNAMP_NB2_61_Mml TAACCATGTAGGAAGAGTTTGAGGTCAGACATGACATTCAGACTGATGTCC**TCAAAACTGAGGGGCATTTTCT**GTGATTTGAAAGGAAAGTGCACCCAGTTTTGGGGATGTCAATTGTGAATACTCA

MYCNAMP_NB2_61_Hsa

UAACCACGUAGGAAGAGUUUGAAGUCAGACAUGACAUUCAGACUGAGGUCC**UCAAAACUGAGGGGCAUUUUCU**GUGGUUUGAAAGGAAAGUGCACCCAGUUUUGGGGAUGUCAAUUGUGAAUCCUCA

.........((((..((((((((((((....)))).))))))))((.(((((((((((((.((.(((((((((...........))))))))).))))))))))))))).)).........)))).. (-52.40)

MYCNAMP_NB2_61_Ptr

UAACCACGUAGGAAGAGUUUGAAGUCAGACGUGACAUUCAGACUGAGGUCC**UCAAAACUGAGGGGCAUUUUCU**GUGGUUUGAAAGGAAAGUGCACCCAGUUUUGGGGAUGUCAAUUGUGAAUACUCA

....((((.......((((((((((((....)))).))))))))((.(((((((((((((.((.(((((((((...........))))))))).))))))))))))))).))...))))........ (-50.70)

MYCNAMP_NB2_61_Mml

UAACCAUGUAGGAAGAGUUUGAGGUCAGACAUGACAUUCAGACUGAUGUCC**UCAAAACUGAGGGGCAUUUUCU**GUGAUUUGAAAGGAAAGUGCACCCAGUUUUGGGGAUGUCAAUUGUGAAUACUCA

...((.....))...((((((((((((....)))).))))))))((((((((((((((((.((.(((((((((...........))))))))).))))))))))))))))))............... (-50.00)

MYCNAMP_NB2_61_Hsa .........---(((-(---..----((((((((((((....)))).))))))))((.((((**(((((((((.((.(((((((((**...........))))))))).))))))))))))))).)).........))))..

MYCNAMP_NB2_61_Ptr ....-----------((((.......((((((((((((....)))).))))))))((.((((**(((((((((.((.(((((((((**...........))))))))).))))))))))))))).))...))))........

MYCNAMP_NB2_61_Mml ...((.....)).---.----.----((((((((((((....)))).))))))))(((((((**(((((((((.((.(((((((((**...........))))))))).))))))))))))))))))...............

------------------------------------------------------------------------------------------------------------------------------------------------------------------------------------------------

-------------

MYCNAMP_NB2_5

-------------

Identity: Ptr 1

**AAGGAGCTTACAATCTAGCTGGG**

MYCNAMP_NB2_5_Hsa AGAAACCTAACCCCCATGGTTGGCGAGGGACTGCTGTGTGTGAAATGGTAACTGCCCTC**AAGGAGCTTACAATCTAGCTGGG**GGTAAATGACTTGCACATGAACACAACTAGACTGTGAGCTTCTAGAGGGCA

MYCNAMP_NB2_5_Ptr AGAAACCTAACCCCCATGGTTGGCGAGGGACTGCTGTGTGTGAAATGGTAACTGCCCTC**AAGGAGCTTACAATCTAGCTGGG**GGTAAATGACTTGCACATGAACACAACTAGACTGTGAGCTTCTAGAGGGCA

MYCNAMP_NB2_5_Hsa

AGAAACCUAACCCCCAUGGUUGGCGAGGGACUGCUGUGUGUGAAAUGGUAACUGCCCUC**AAGGAGCUUACAAUCUAGCUGGG**GGUAAAUGACUUGCACAUGAACACAACUAGACUGUGAGCUUCUAGAGGGCA

..........((((((....)))...)))..((((((.......))))))...((((((.(((((((((((.(((((.((...((..(((.......)))..)).)).))))).))))))))))).)))))). (-48.30)

MYCNAMP_NB2_5_Ptr

AGAAACCUAACCCCCAUGGUUGGCGAGGGACUGCUGUGUGUGAAAUGGUAACUGCCCUC**AAGGAGCUUACAAUCUAGCUGGG**GGUAAAUGACUUGCACAUGAACACAACUAGACUGUGAGCUUCUAGAGGGCA

..........((((((....)))...)))..((((((.......))))))...((((((.(((((((((((.(((((.((...((..(((.......)))..)).)).))))).))))))))))).)))))). (-48.30)

MYCNAMP_NB2_5_Hsa ..........((((((....)))...)))..((((((.......))))))...((((((**.(((((((((((.(((((.((..**.((..(((.......)))..)).)).))))).))))))))))).))))))

MYCNAMP_NB2_5_Ptr ..........((((((....)))...)))..((((((.......))))))...((((((**.(((((((((((.(((((.((..**.((..(((.......)))..)).)).))))).))))))))))).))))))

------------------------------------------------------------------------------------------------------------------------------------------------------------------------------------------------

--------------

MYCNSC_NB5_281

--------------

Identity: Ptr 1 Mml 0,99 Cfa 0,98 Bta 0,95

TCCATTACACTACCCTGCCTCT

MYCNSC_NB5_281_Hsa GACGACACCGTGACTGCAG-AAGGCCCAGTCTGCTACTCGGCCCGCACTCTC**TCCATTACACTACCCTGCCTCT**TCTCCATGAGAGGCAGCGGGGTGTAGTGGATAGAGCACGGGTTCAAGTCCCG

MYCNSC_NB5_281_Ptr GACGACACCGTGACTGCAG-AAGGCCCAGTCTGCTACTCGGCCCGCACTCTC**TCCATTACACTACCCTGCCTCT**TCTCCATGAGAGGCAGCGGGGTGTAGTGGATAGAGCACGGGTTCAAGTCCCG

MYCNSC_NB5_281_Mml GACGACACCGTGACTGCAG-AAGGCCCAGTATGCTACTCGGCCCGCACTCTC**TCCATTACACTACCCTGCCTCT**TCTCCATGAGAGGCAGCGGGGTGTAGTGGATAGAGCACGGGTTCAAGTCCCG

MYCNSC_NB5_281_Cfa GACGACACCGTGACTGCAGGAAGGCCCAGTCTGCCGCTCGGCCCGCGCTCTC**TCCATTACACTACCCTGCCTCT**TCTCCATGAGAGGCAGCGGGGTGTAGTGGATAGAGCACGGGTTCAAGTCCCG

MYCNSC_NB5_281_Bta GACGACACCGTGACTGCAGGGAGGCCCCGTCTGCCACGCGGCCCGCGCTCTC**TCCATTACACTACCCTGCCTCT**TCTCCACGAGAGGCAGCGGGGTGTAGTGGATAGAGCACGGGTTCAAGTCCCG

MYCNSC_NB5_281_Hsa

GACGACACCGUGACUGCAGAAGGCCCAGUCUGCUACUCGGCCCGCACUCUC**UCCAUUACACUACCCUGCCUCU**UCUCCAUGAGAGGCAGCGGGGUGUAGUGGAUAGAGCACGGGUUCAAGUCCCG

........((.(((((((((........))))).....((((((..((((.(((((((((((.(((((((((((......))))))))).))))))))))))).))))..))))))..)))).)) (-59.40)

MYCNSC_NB5_281_Ptr

GACGACACCGUGACUGCAGAAGGCCCAGUCUGCUACUCGGCCCGCACUCUC**UCCAUUACACUACCCUGCCUCU**UCUCCAUGAGAGGCAGCGGGGUGUAGUGGAUAGAGCACGGGUUCAAGUCCCG

........((.(((((((((........))))).....((((((..((((.(((((((((((.(((((((((((......))))))))).))))))))))))).))))..))))))..)))).)) (-59.40)

MYCNSC_NB5_281_Mml

GACGACACCGUGACUGCAGAAGGCCCAGUAUGCUACUCGGCCCGCACUCUC**UCCAUUACACUACCCUGCCUCU**UCUCCAUGAGAGGCAGCGGGGUGUAGUGGAUAGAGCACGGGUUCAAGUCCCG

((((((.(((((...((....((((.((((...)))).)))).)).((((.(((((((((((.(((((((((((......))))))))).))))))))))))).))))))))))))...)))... (-59.80)

MYCNSC_NB5_281_Cfa

GACGACACCGUGACUGCAGGAAGGCCCAGUCUGCCGCUCGGCCCGCGCUCUC**UCCAUUACACUACCCUGCCUCU**UCUCCAUGAGAGGCAGCGGGGUGUAGUGGAUAGAGCACGGGUUCAAGUCCCG

........((.((((((.((.((((...)))).))))..((((((.(((((.(((((((((((.(((((((((((......))))))))).))))))))))))).))))).))))))..)))).)) (-64.10)

MYCNSC_NB5_281_Bta

GACGACACCGUGACUGCAGGGAGGCCCCGUCUGCCACGCGGCCCGCGCUCUC**UCCAUUACACUACCCUGCCUCU**UCUCCACGAGAGGCAGCGGGGUGUAGUGGAUAGAGCACGGGUUCAAGUCCCG

(((....(((((...((((((....)))...)))..)))))((((.(((((.(((((((((((.(((((((((((......))))))))).))))))))))))).))))).)))).....)))... (-66.80)

MYCNSC_NB5_281_Hsa ........((.-(((--------(-((-((-(.....-.-..-)---)))).....((((((..((((.**(((((((((((.((((((((((**(......))))))))).))))))))))))).))))..))))))..)))).))----------

MYCNSC_NB5_281_Ptr ........((.-(((--------(-((-((-(.....-.-..-)---)))).....((((((..((((.**(((((((((((.((((((((((**(......))))))))).))))))))))))).))))..))))))..)))).))----------

MYCNSC_NB5_281_Mml ((((----((.(((((...((....((-((.((((..-.)))-)--.)))).-)-)-------.((((.**(((((((((((.((((((((((**(......))))))))).))))))))))))).))))--------)--)))))))...)))...

MYCNSC_NB5_281_Cfa ........((.-(((--------(-((.((.((((..-.)))-)--.)))).-.--((((((.(((((.**(((((((((((.((((((((((**(......))))))))).))))))))))))).))))).))))))..)))).))----------

MYCNSC_NB5_281_Bta ------------(((....((-((-(...((((((....)))...)))..))-)))--((((.(((((.**(((((((((((.((((((((((**(......))))))))).))))))))))))).))))).)))).....)))...----------

------------------------------------------------------------------------------------------------------------------------------------------------------------------------------------------------

-------------

CONTIG_CHR_9

-------------

Identity: Ptr 1 Mml 1 Mdo 0,82 Mmu 0,84 Rno 0,86 Cfa 0,95 Bta 0,90

TGCAGGAACTTGTGAGTCTCC

CONTIG_CHR_9_Hsa GAACAGCTGCTTATCTGCAAATGGTTCCTGCTTAGAA-TAAGTTTGTGTGCATT**TGCAGGAACTTGTGAGTCTCC**TATTGAAAATGAACAGGAGACTGATGAGTTCCCGGGAACACCCACAAATCTT

CONTIG_CHR_9_Ptr GAACAGCTGCTTATCTGCAAATGGTTCCTGCTTAGAA-TAAGTTTGTGTGCATT**TGCAGGAACTTGTGAGTCTCC**TATTGAAAATGAACAGGAGACTGATGAGTTCCCGGGAACACCCACAAATCTT

CONTIG_CHR_9_Mml GAACAGCTGCTTATCTGCAAATGGTTCCTGCTTAGAA-TAAGTTTGTGTGCATT**TGCAGGAACTTGTGAGTCTCC**TATTGAAAATGAACAGGAGACTGATGAGTTCCCGGGAACACCCACAAATCTT

CONTIG_CHR_9_Mdo CTGTTCCTGCCTGCCAAACATGGGTACCAGCTTTAAA-CAAGTTTGTGTGCATT**TGCAGGAACTTGTGAGTCTCC**TATTGAAAATGAACGGGAGACTGGTGAGTTCCCGGGAACACCCACAAATTTT

CONTIG_CHR_9_Mmu CAGCATCTCCTTAGGAACAACGAGCACCAGCTTAAAA-TCAGTTTGTGTGCATT**TGCAGGAACTTGTGAGTCTCC**TATTGAAAATAGACAGGAGACTGACAAGTTCCCGGGAACACCCACAAATCTT

CONTIG_CHR_9_Rno CAGCATCTTCTTATGAACAACGAACACCTGCTTAAAA-TCAGTTTGTGTGCATT**TGCAGGAACTTGTGAGTCTCC**TATTGAAAATGGACAGGAGACTGACAAGTTCCCGGGAACACCCACAAATCTT

CONTIG_CHR_9_Cfa CAACATCTGCTTATCTGCCAAGGGTGCCTGCTTAAAAATAAGTTTGTGTGCATT**TGCAGGAACTTGTGAGTCTCC**TATTGAAAATGAACAGGAGACTGATGAGTTCCCGGGAACACCCACAAATCTT

CONTIG_CHR_9_Bta CAGCATCTGCCTCTCTGCCAAGGGTGCCTGCTTAC---TGAGTTTGTGTGCATT**TGCAGGAACTTGTGAGTCTCC**TATTGAACATGAACAGGAGGCTGATGAGTTCCCGGGAACACCCACAAATCTT

CONTIG_CHR_9_Hsa

GAACAGCUGCUUAUCUGCAAAUGGUUCCUGCUUAGAAUAAGUUUGUGUGCAUU**UGCAGGAACUUGUGAGUCUCC**UAUUGAAAAUGAACAGGAGACUGAUGAGUUCCCGGGAACACCCACAAAUCUU

...((..(((......)))..))(((((((.................(((....)))(((((((((.((((((((.((.......)).)))))))).))))))))))))))))............. (-38.80)

CONTIG_CHR_9_Ptr

GAACAGCUGCUUAUCUGCAAAUGGUUCCUGCUUAGAAUAAGUUUGUGUGCAUU**UGCAGGAACUUGUGAGUCUCC**UAUUGAAAAUGAACAGGAGACUGAUGAGUUCCCGGGAACACCCACAAAUCUU

...((..(((......)))..))(((((((.................(((....)))(((((((((.((((((((.((.......)).)))))))).))))))))))))))))............. (-38.80)

CONTIG_CHR_9_Mml

GAACAGCUGCUUAUCUGCAAAUGGUUCCUGCUUAGAAUAAGUUUGUGUGCAUU**UGCAGGAACUUGUGAGUCUCC**UAUUGAAAAUGAACAGGAGACUGAUGAGUUCCCGGGAACACCCACAAAUCUU

...((..(((......)))..))(((((((.................(((....)))(((((((((.((((((((.((.......)).)))))))).))))))))))))))))............. (-38.80)

CONTIG_CHR_9_Mdo

CUGUUCCUGCCUGCCAAACAUGGGUACCAGCUUUAAACAAGUUUGUGUGCAUU**UGCAGGAACUUGUGAGUCUCC**UAUUGAAAAUGAACGGGAGACUGGUGAGUUCCCGGGAACACCCACAAAUUUU

.((((((((....(((....))).((((........((((((((.(((......))).)))))))).(((((((..((.......))..)))))))))))......))))))))............ (-MYCNSC_NB3.20)

CONTIG_CHR_9_Mmu

CAGCAUCUCCUUAGGAACAACGAGCACCAGCUUAAAAUCAGUUUGUGUGCAUU**UGCAGGAACUUGUGAGUCUCC**UAUUGAAAAUAGACAGGAGACUGACAAGUUCCCGGGAACACCCACAAAUCUU

..(((..(((...))).......(((((((((.......)).))).))))...))).(((((((((.((((((((.((.......)).)))))))).))))))))).(((....)))......... (-42.50)

CONTIG_CHR_9_Rno

CAGCAUCUUCUUAUGAACAACGAACACCUGCUUAAAAUCAGUUUGUGUGCAUU**UGCAGGAACUUGUGAGUCUCC**UAUUGAAAAUGGACAGGAGACUGACAAGUUCCCGGGAACACCCACAAAUCUU

..(((..(((....)))......((((..(((.......)))..)))).....))).(((((((((.((((((((.((.......)).)))))))).))))))))).(((....)))......... (-MYCNSC_NB3.20)

CONTIG_CHR_9_Cfa

CAACAUCUGCUUAUCUGCCAAGGGUGCCUGCUUAAAAAUAAGUUUGUGUGCAUU**UGCAGGAACUUGUGAGUCUCC**UAUUGAAAAUGAACAGGAGACUGAUGAGUUCCCGGGAACACCCACAAAUCUU

........((......))...(((((((((..................(((....)))(((((((((.((((((((.((.......)).)))))))).)))))))))))))..)))))......... (-MYCNSC_NB3.10)

CONTIG_CHR_9_Bta

CAGCAUCUGCCUCUCUGCCAAGGGUGCCUGCUUACUGAGUUUGUGUGCAUU**UGCAGGAACUUGUGAGUCUCC**UAUUGAACAUGAACAGGAGGCUGAUGAGUUCCCGGGAACACCCACAAAUCUU

..(((...((..((((....)))).)).(((.(((.......))).)))..))).(((((((((.((((((((.((.......)).)))))))).))))))))).(((....)))......... (-MYCNSC_NB3.70)

CONTIG_CHR_9_Hsa --...---((..(((......)))..))--(((((((.-.-------.----.--..--.-..........((--(.-..**.)))**-----------------**(((((((((.(((((((**(.((.......)).)))))))).)))))))))))))---)------)

CONTIG_CHR_9_Ptr --...---((..(((......)))..))--(((((((.-.-------.----.--..--.-..........((--(.-..**.)))**-----------------**(((((((((.(((((((**(.((.......)).)))))))).)))))))))))))---)------)

CONTIG_CHR_9_Mml --...---((..(((......)))..))--(((((((.-.-------.----.--..--.-..........((--(.-..**.)))**-----------------**(((((((((.(((((((**(.((.......)).)))))))).)))))))))))))---)------)

CONTIG_CHR_9_Mdo .((((((((....(((....)))-------.--((((.-.-------.----.--..--.-.((((((((.(((...-..**.)))**-**.**-**)))))**---**)))**---**.**---------**(((((((**..((.......))..))))))---)--)-----)))---..-----.

CONTIG_CHR_9_Mmu --------------.--.------------------(((..------(((...-))).......-(((((((((.......)).))).))))--...**))).(((((((((.(((((((**(.((.......)).)))))))).)))))))))---.(((....)))-

CONTIG_CHR_9_Rno --------------.--.------------------(((..------(((....)))-......-((((..(((.......)))-.-.)))).....**))).(((((((((.(((((((**(.((.......)).)))))))).)))))))))---.(((....)))-

CONTIG_CHR_9_Cfa --...---.....((......))...--(((((((((.-.-------.----.--..--............((--(.-..**.)))**-----------------**(((((((((.(((((((**(.((.......)).)))))))).)))))))))))))---..----))

CONTIG_CHR_9_Bta --------------.--.------------------(((...((..((((....)))-).)).--((-(-.(((.......)))---.)-))---..**))).(((((((((.(((((((**(.((.......)).)))))))).)))))))))---.(((....)))-

CONTIG_CHR_9_Hsa )...-------.--.........

CONTIG_CHR_9_Ptr )...-------.--.........

CONTIG_CHR_9_Mml )...-------.--.........

CONTIG_CHR_9_Mdo ...))))))))............

CONTIG_CHR_9_Mmu --------------.........

CONTIG_CHR_9_Rno --------------.........

CONTIG_CHR_9_Cfa )-))----------.........

CONTIG_CHR_9_Bta --------------.........

------------------------------------------------------------------------------------------------------------------------------------------------------------------------------------------------

--------------

MYCNSC_NB5_318

--------------

Identity: Ptr 1 Mml 1 Mmu 0,80 Rno 0,83 Cfa 0,96 Bta 0,92

TGGATTTCTTTGTGAATCA

MYCNSC_NB5_318_Hsa GGCCAAAGGGTACTGTTTTTGTTGGTTCATAGGTACCAGGCTTTACACAAACTGTGAAGTGCTG**TGGATTTCTTTGTGAATCA**CCATATCTAAGCTAATGTGGTGGTGGTTTACAAAGTAATTCATAGTGCTTC

MYCNSC_NB5_318_Ptr GGCCAAAGGGTACTGTTTTTGTTGGTTCATAGGTACCAGGCTTTACACAAACTGTGAAGTGCTG**TGGATTTCTTTGTGAATCA**CCATATCTAAGCTAATGTGGTGGTGGTTTACAAAGTAATTCATAGTGCTTC

MYCNSC_NB5_318_Mml GGCCAAAGGGTACTGTTTTTGTTGGTTCATAGGTACCAGGCTTTACACAAACTGTGAAGTGCTG**TGGATTTCTTTGTGAATCA**CCATATCTAAGCTAATGTGGTGGTGGTTTACAAAGTAATTCATAGTGCTTC

MYCNSC_NB5_318_Mmu CATGGTAGCATATAATTTTTAT--GTTCATGTGTAC-AAGCATTACATAAACTGTCACGTGCTA**TGGATTTCTCTGTGAATCA**CTATATCAAATCTAGTGTGGTAGTGGTTTACAAAGTAATTCATAGTGCTTC

MYCNSC_NB5_318_Rno CATGGTAGCATATGATTTTTATTAGTTCATGGATAC-AAGCATTACATAAACTGTCAAGTGCTG**TGGATTTCTCTGTGAATCA**CCATATCAAATCTAATGTGGTGGTGGTTTGCAAAGTAATTCATAGTGCTTC

MYCNSC_NB5_318_Cfa GGTATAAGGGTACTGTTTTTATTGGTTCATAGGTACCAAGCTTTACCCAAACTGTGAAGTGCTG**TGGATTTCTTTGTGAATCA**CCATATCTAAGCTAATGTGGTGGTGGTTTACAAAGTAATTCATAGTGCTTC

MYCNSC_NB5_318_Bta GGTATACAGGTGCTGTTTTTATTGGTTCATAGGTACCAAGCTGTACCCAAACTGTGAAGTGCTG**TGGATTTCTTTGTGAATCA**CCATATCTAAGCTAATGTGGTGGTGGTTTACAAAGTAATTCATAGTGCTTC

MYCNSC_NB5_318_Hsa

GGCCAAAGGGUACUGUUUUUGUUGGUUCAUAGGUACCAGGCUUUACACAAACUGUGAAGUGCUG**UGGAUUUCUUUGUGAAUCA**CCAUAUCUAAGCUAAUGUGGUGGUGGUUUACAAAGUAAUUCAUAGUGCUUC

((((....((((((.....((......))..)))))).)))).............((((..(((((((((.((((((((((((((((......((....)).)))))))))))))))).)))))))))..)))) (-53.20)

MYCNSC_NB5_318_Ptr

GGCCAAAGGGUACUGUUUUUGUUGGUUCAUAGGUACCAGGCUUUACACAAACUGUGAAGUGCUG**UGGAUUUCUUUGUGAAUCA**CCAUAUCUAAGCUAAUGUGGUGGUGGUUUACAAAGUAAUUCAUAGUGCUUC

((((....((((((.....((......))..)))))).)))).............((((..(((((((((.((((((((((((((((......((....)).)))))))))))))))).)))))))))..)))) (-53.20)

MYCNSC_NB5_318_Mml

GGCCAAAGGGUACUGUUUUUGUUGGUUCAUAGGUACCAGGCUUUACACAAACUGUGAAGUGCUG**UGGAUUUCUUUGUGAAUCA**CCAUAUCUAAGCUAAUGUGGUGGUGGUUUACAAAGUAAUUCAUAGUGCUUC

((((....((((((.....((......))..)))))).)))).............((((..(((((((((.((((((((((((((((......((....)).)))))))))))))))).)))))))))..)))) (-53.20)

MYCNSC_NB5_318_Mmu

CAUGGUAGCAUAUAAUUUUUAUGUUCAUGUGUACAAGCAUUACAUAAACUGUCACGUGCUA**UGGAUUUCUCUGUGAAUCA**CUAUAUCAAAUCUAGUGUGGUAGUGGUUUACAAAGUAAUUCAUAGUGCUUC

..((..((.........(((((((..((((......)))).)))))))))..)).((((((((((((.((.(((((((((((((..((.......))..))))))))))))).)).))))))))))))... (-38.50)

MYCNSC_NB5_318_Rno

CAUGGUAGCAUAUGAUUUUUAUUAGUUCAUGGAUACAAGCAUUACAUAAACUGUCAAGUGCUG**UGGAUUUCUCUGUGAAUCA**CCAUAUCAAAUCUAAUGUGGUGGUGGUUUGCAAAGUAAUUCAUAGUGCUUC

....(((...(((((...........)))))..)))...................(((..(((((((((.((.((..(((((((((..((.......))..)))))))))..)).)).)))))))))..))). (-38.80)

MYCNSC_NB5_318_Cfa

GGUAUAAGGGUACUGUUUUUAUUGGUUCAUAGGUACCAAGCUUUACCCAAACUGUGAAGUGCUG**UGGAUUUCUUUGUGAAUCA**CCAUAUCUAAGCUAAUGUGGUGGUGGUUUACAAAGUAAUUCAUAGUGCUUC

.......(((((..((.....(((((........)))))))..))))).......((((..(((((((((.((((((((((((((((......((....)).)))))))))))))))).)))))))))..)))) (-53.30)

MYCNSC_NB5_318_Bta

GGUAUACAGGUGCUGUUUUUAUUGGUUCAUAGGUACCAAGCUGUACCCAAACUGUGAAGUGCUG**UGGAUUUCUUUGUGAAUCA**CCAUAUCUAAGCUAAUGUGGUGGUGGUUUACAAAGUAAUUCAUAGUGCUUC

((((((..((((((.................))))))....))))))........((((..(((((((((.((((((((((((((((......((....)).)))))))))))))))).)))))))))..)))) (-52.13)

MYCNSC_NB5_318_Hsa ----(-----(((....-((((((.....((.--.....))..-)))))).----)---))).............((((..(((**((((((.((((((((((((**((((......((....))--.)))))))))))))))).)))))))))..))))

MYCNSC_NB5_318_Ptr ----(-----(((....-((((((.....((.--.....))..-)))))).----)---))).............((((..(((**((((((.((((((((((((**((((......((....))--.)))))))))))))))).)))))))))..))))

MYCNSC_NB5_318_Mml ----(-----(((....-((((((.....((.--.....))..-)))))).----)---))).............((((..(((**((((((.((((((((((((**((((......((....))--.)))))))))))))))).)))))))))..))))

MYCNSC_NB5_318_Mmu ..((..((.........(((((((-..((((.--.....)))).)))))))---------))----.-.--))--.--((((((**((((((.((.(((((((((**((((..((.......--))..))))))))))))).)).))))))))))))...

MYCNSC_NB5_318_Rno ----.-.---..(((..-.(((((-.......--....)------))))..---)))...................(((..(((**((((((.((.((..(((((**((((..((.......--))..)))))))))..)).)).)))))))))..))).

MYCNSC_NB5_318_Cfa ---.......(((((..--((.....(((((........))))-))--)..---)))--))-----.-..-....((((..(((**((((((.((((((((((((**((((......((....))--.)))))))))))))))).)))))))))..))))

MYCNSC_NB5_318_Bta ----(-----(((((..-((((((........--.........-))))))....)))--)))---..-..-....((((..(((**((((((.((((((((((((**((((......((....))--.)))))))))))))))).)))))))))..))))

------------------------------------------------------------------------------------------------------------------------------------------------------------------------------------------------

--------------

MYCNSC_NB5_330

--------------

Identity: Ptr 1 Mml 1 Mmu 0,95 Rno 0,95 Cfa 0,98 Bta 0,96

TAGGACACATGGTCTACTTCT

MYCNSC_NB5_330_Hsa ACTTCCTGGTATTTGAAGATGCGGTTGACCATGGTGTGTACGCTTTATTTGTGACG**TAGGACACATGGTCTACTTCT**TCTCAATATCACATCT-CGCCTTGGAAGAC-TTCCAGGAGGTGATATCAGCTTTG

MYCNSC_NB5_330_Ptr ACTTCCTGGTATTTGAAGATGCGGTTGACCATGGTGTGTACGCTTTATTTGTGACG**TAGGACACATGGTCTACTTCT**TCTCAATATCACATCT-CGCCTTGGAAGAC-TTCCAGGAGGTGATATCAGCTTTG

MYCNSC_NB5_330_Mml ACTTCCTGGTATTTGAAGATGCGGTTGACCATGGTGTGTACGCTTTATTTATAACG**TAGGACACATGGTCTACTTCT**TCTCAATATCACATCT-CGCCTCGGAAGAC-TTCCAGGAGGTGATATCAGCTTTG

MYCNSC_NB5_330_Mmu ACCTCAAGGTATTTGAAGATGCGGTTGACCATGGTGTGTACGCTTTATTTATGACG**TAGGACACATGGTCTACTTCT**TCTCAATATCACATCT-CGCCTTGGAAGAC-TTCCAGGAGGTGACATCTGCTTTG

MYCNSC_NB5_330_Rno ACCTCATGGTATTTGAAGGTGCGGTTGACCATGGTGTGTACGCTTTATTTATGACA**TAGGACACATGGTCTACTTCT**TCTCAATATCACATCT-CGCCTTGGAAGAC-TTCCAGGAGGTGACATCTGCTTTG

MYCNSC_NB5_330_Cfa ACTTCCTGGTATTTGAAGATGCGGTTGACCATGGTGTGTACGCTTTATTTATGACG**TAGGACACATGGTCTACTTCT**TCTCAATATCACATCT-CGCCTCGGAAGAC-TTCCAGGAGGTGATATCAGCTTTG

MYCNSC_NB5_330_Bta --TTCCTGGTATTTGAAGACGCGGTTGACCATGGTGTGTACGCTTTATTTATGACG**TAGGACACATGGTCTACTTCT**TCTCGATATCACATCTTCGCCTTGGAAGACCTTCCTGGAGGTGATCTCAGCTTTG

MYCNSC_NB5_330_Hsa

ACUUCCUGGUAUUUGAAGAUGCGGUUGACCAUGGUGUGUACGCUUUAUUUGUGACG**UAGGACACAUGGUCUACUUCU**UCUCAAUAUCACAUCUCGCCUUGGAAGACUUCCAGGAGGUGAUAUCAGCUUUG

......(((((((.(((((...(((.(((((((.(((.((((.((.......))))))..)))))))))).))))))))..)))))))....(((((((((((...)))))..))))))........... (-36.00)

MYCNSC_NB5_330_Ptr

ACUUCCUGGUAUUUGAAGAUGCGGUUGACCAUGGUGUGUACGCUUUAUUUGUGACG**UAGGACACAUGGUCUACUUCU**UCUCAAUAUCACAUCUCGCCUUGGAAGACUUCCAGGAGGUGAUAUCAGCUUUG

......(((((((.(((((...(((.(((((((.(((.((((.((.......))))))..)))))))))).))))))))..)))))))....(((((((((((...)))))..))))))........... (-36.00)

MYCNSC_NB5_330_Mml

ACUUCCUGGUAUUUGAAGAUGCGGUUGACCAUGGUGUGUACGCUUUAUUUAUAACG**UAGGACACAUGGUCUACUUCU**UCUCAAUAUCACAUCUCGCCUCGGAAGACUUCCAGGAGGUGAUAUCAGCUUUG

......(((((((.(((((...(((.(((((((.(((.((((............))))..)))))))))).))))))))..)))))))....(((((((((((...))))..)))))))........... (-37.10)

MYCNSC_NB5_330_Mmu

ACCUCAAGGUAUUUGAAGAUGCGGUUGACCAUGGUGUGUACGCUUUAUUUAUGACG**UAGGACACAUGGUCUACUUCU**UCUCAAUAUCACAUCUCGCCUUGGAAGACUUCCAGGAGGUGACAUCUGCUUUG

....(((((((...(((((...(((.(((((((.(((.((((.((.......))))))..)))))))))).)))))))).........((((((...((((((...)))))))))))).....))))))) (-36.10)

MYCNSC_NB5_330_Rno

ACCUCAUGGUAUUUGAAGGUGCGGUUGACCAUGGUGUGUACGCUUUAUUUAUGACA**UAGGACACAUGGUCUACUUCU**UCUCAAUAUCACAUCUCGCCUUGGAAGACUUCCAGGAGGUGACAUCUGCUUUG

......(((((((.(((((...(((.(((((((((((((..............))))...)).))))))).))))))))..)))))))....(((((((((((...)))))..))))))........... (-32.64)

MYCNSC_NB5_330_Cfa

ACUUCCUGGUAUUUGAAGAUGCGGUUGACCAUGGUGUGUACGCUUUAUUUAUGACG**UAGGACACAUGGUCUACUUCU**UCUCAAUAUCACAUCUCGCCUCGGAAGACUUCCAGGAGGUGAUAUCAGCUUUG

......(((((((.(((((...(((.(((((((.(((.((((.((.......))))))..)))))))))).))))))))..)))))))....(((((((((((...))))..)))))))........... (-37.30)

MYCNSC_NB5_330_Bta

UUCCUGGUAUUUGAAGACGCGGUUGACCAUGGUGUGUACGCUUUAUUUAUGACG**UAGGACACAUGGUCUACUUCU**UCUCGAUAUCACAUCUUCGCCUUGGAAGACCUUCCUGGAGGUGAUCUCAGCUUUG

....(((((((.(((((...(((.(((((((.(((.((((.((.......))))))..)))))))))).))))))))..))))))).....(((((((((((....))))..)))))))........... (-38.00)

MYCNSC_NB5_330_Hsa ......----(((((((.(((((...(((.(((((((.(((.((((.((.......))))**))--..)))))))))).))))))**))..)))))))....-((((((----(((((...-)))))..))))))-...........

MYCNSC_NB5_330_Ptr ......----(((((((.(((((...(((.(((((((.(((.((((.((.......))))**))--..)))))))))).))))))**))..)))))))....-((((((----(((((...-)))))..))))))-...........

MYCNSC_NB5_330_Mml ......----(((((((.(((((...(((.(((((((.(((.((((............))**))--..)))))))))).))))))**))..)))))))....-((((((----(((((...-))))..)))))))-...........

MYCNSC_NB5_330_Mmu ....((((((----(...(((((...(((.(((((((.(((.((((.((.......))))**))--..)))))))))).))))))**)).......--..---((((((...((((((...-))))))-)))))).....)))))))

MYCNSC_NB5_330_Rno ......----(((((((.(((((...(((.(((((((--((((((..............)))**)...)).))))))).))))))**))..)))))))....-((((((----(((((...-)))))..))))))-...........

MYCNSC_NB5_330_Cfa ......----(((((((.(((((...(((.(((((((.(((.((((.((.......))))**))--..)))))))))).))))))**))..)))))))....-((((((----(((((...-))))..)))))))-...........

MYCNSC_NB5_330_Bta --....----(((((((.(((((...(((.(((((((.(((.((((.((.......))))**))--..)))))))))).))))))**))..))))))).....((((((----(((((....))))..)))))))-...........

------------------------------------------------------------------------------------------------------------------------------------------------------------------------------------------------

--------------

MYCNSC_NB2_237

--------------

Identity: Ptr 0,99 Mml 0,94 Mmu 0,62 Rno 0,61 Cfa 0,6 Bta 0,67

**GAT-GATGCTGCTGA---TGCTG**

MYCNSC_NB2_237_Hsa AGTATCATG---AATTAGAAAC--CTACTTATTACAT-AGTTTACATAAGAAGCGT**GAT-GATGCTGCTGA---TGCTG**TAATATCTAGTCTCTGT-TGATGGTTCTTTCCTGGGAGGTTGGATGTGTTT

MYCNSC_NB2_237_Ptr AGTATCATG---AATTAGAAAC--CTACTTATTACAT-AGTTTACATAAGAAGCGT**GAT-GATGCTGCTGA---TGCTG**TGATATCTAGTCTCTGT-TGATGGTTCTTTCCTGGGAGGTTGGATGTGTTT

MYCNSC_NB2_237_Mml AGTATCATG---AATTAGAAAC--CTACTTATTACAT-AGTATACATAAGAAGCAT**GAT-GATGATGCTGA---TGCTG**TCATATCTAGTCTCTAC-CGATGGTTCTTTCCTGGGAGGTTGGATGTGTTT

MYCNSC_NB2_237_Mmu ------------GGCCACAAGC---TATTTATTACAT-GCTGTATTTCGTGTGCGC**TAGGTGTGATGGTTACTGCTCTG**TGGTATCCATACACTATTTAATTGTTCTT---TGGGAAATTGGATGTCTGT

MYCNSC_NB2_237_Rno ------------GGCCACAAGC---TATTTCTTACAT-GCTGTACTTCGTGTGTGC**GAGGTGTAATGGTTACTGTTCTG**CAGTATCCATTCACTCTTTGATTGTTCTT---TGGGAAACTGAATGACTGT

MYCNSC_NB2_237_Cfa AGTACACTTTCCAGGTAAAAGC--CTACTTATTACAT-ACTACAAGTAT-AAATAT**GAA-TGTAATCCTAT---TGTGG**T--AATCTAGATACCATATGATGGCTCTTCTATGGGACACGCCTTTTTTTT

MYCNSC_NB2_237_Bta AGCATACTTCCCAGGTATAAACCCCTGTTTATCATATTAATATACTTA--AAGTAT**AAG-TATGATGATAA---TTATG**T--TATCTAGTCACCTTGTGATGGTTCATCTATGGAAGACTGGATGTATAT

MYCNSC_NB2_237_Hsa

AGUAUCAUGAAUUAGAAACCUACUUAUUACAUAGUUUACAUAAGAAGCGU**GAUGAUGCUGCUGAUGCUG**UAAUAUCUAGUCUCUGUUGAUGGUUCUUUCCUGGGAGGUUGGAUGUGUUU

(((((((...((((...((((.(((((............))))).)).))..)))).....)))))))...(((((((..((((...((........))...))))..))))))).... (-24.60)

MYCNSC_NB2_237_Ptr

AGUAUCAUGAAUUAGAAACCUACUUAUUACAUAGUUUACAUAAGAAGCGU**GAUGAUGCUGCUGAUGCUG**UGAUAUCUAGUCUCUGUUGAUGGUUCUUUCCUGGGAGGUUGGAUGUGUUU

(((((((...((((...((((.(((((............))))).)).))..)))).....)))))))...(((((((..((((...((........))...))))..))))))).... (-24.70)

MYCNSC_NB2_237_Mml

AGUAUCAUGAAUUAGAAACCUACUUAUUACAUAGUAUACAUAAGAAGCAU**GAUGAUGAUGCUGAUGCUG**UCAUAUCUAGUCUCUACCGAUGGUUCUUUCCUGGGAGGUUGGAUGUGUUU

((((((..............((((........)))).........(((((.......)))))))))))..((((((((..((((...((........))...))))..))))))))... (-27.50)

MYCNSC_NB2_237_Mmu

GGCCACAAGCUAUUUAUUACAUGCUGUAUUUCGUGUGCGC**UAGGUGUGAUGGUUACUGCUCUG**UGGUAUCCAUACACUAUUUAAUUGUUCUUUGGGAAAUUGGAUGUCUGU

.(((((((((...(((((((((((.((((.....))))))...))))))))).....))).))))))......(((.(((((((((.(((....))))))))))))..))) (-26.30)

MYCNSC_NB2_237_Rno

GGCCACAAGCUAUUUCUUACAUGCUGUACUUCGUGUGUGC**GAGGUGUAAUGGUUACUGUUCUG**CAGUAUCCAUUCACUCUUUGAUUGUUCUUUGGGAAACUGAAUGACUGU

(((.....)))......(((((((........)))))))(((((.(((((((.(((((.....))))).))))).)).)))))...(((.(((((....))))).)))... (-29.50)

MYCNSC_NB2_237_Cfa

AGUACACUUUCCAGGUAAAAGCCUACUUAUUACAUACUACAAGUAUAAAUAU**GAAUGUAAUCCUAUUGUGG**UAAUCUAGAUACCAUAUGAUGGCUCUUCUAUGGGACACGCCUUUUUUUU

........(((((......((((.....(((((((...................))))))).....(((((((.......)))))))....))))......))))).............. (-18.61)

MYCNSC_NB2_237_Bta

AGCAUACUUCCCAGGUAUAAACCCCUGUUUAUCAUAUUAAUAUACUUAAAGUAU**AAGUAUGAUGAUAAUUAUG**UUAUCUAGUCACCUUGUGAUGGUUCAUCUAUGGAAGACUGGAUGUAUAU

.(((((.....((((........)))).((((((((((..(((((.....)))))))))))))))....)))))(((((((((.((....((((...))))...))..)))))))))..... (-30.80)

MYCNSC_NB2_237_Hsa -----(------((((-(---------(--------------...--((((...( (((.-(-((((............))))-).)).))..))))--..----------

MYCNSC_NB2_237_Ptr -----(------((((-(---------(--------------...--((((...( (((.-(-((((............))))-).)).))..))))--..----------

MYCNSC_NB2_237_Mml -----(------((((-(---------.--------------...--........ ..(--(-((-.-.--.--.--....))-))-.........-(-((((.......-

MYCNSC_NB2_237_Mmu .----(------((((((-----(---(--------------...--((((((-( (-(--((.(((-(--.--....)))))-)...)))))))))--..----------

MYCNSC_NB2_237_Rno -(((.....)))....-.-----.(((((((........)))))))-(((((.-( ((((-((.(((-(-(..-...))))).))))).)).)))))--------------

MYCNSC_NB2_237_Cfa -...-...--..(((((......(-------------------(--((.....-( ((((-((..........-.........))))))-).....-(((((((.......

MYCNSC_NB2_237_Bta -----.------(((((....--.--((-((........))-)).--((((((-( (((..(-((((-.--.--.--..))))-))-))-)))))))--------------

MYCNSC_NB2_237_Hsa --...)---------------))))))...---(((((((..((((...((.... ....))...))))..)))))))..-.-.

MYCNSC_NB2_237_Ptr --...)---------------))))))...---(((((((..((((...((.... ....))...))))..)))))))..-.-.

MYCNSC_NB2_237_Mml -)))))---------------))))))..(---(((((((..((((...((.... ....))...))))..)))))))).-.-.

MYCNSC_NB2_237_Mmu --...)--------)-)-.--))))))......(((-.((((((((-(.(((.-. ..))-)-))-)))))))..)))------

MYCNSC_NB2_237_Rno ----..-----------------.---------(((-.(--(-(---(-(--.-. --..-)-)---)-)--).-)))-.-.-.

MYCNSC_NB2_237_Cfa )))))))....))))......)))))--------..-..--.-.---.------. --..---.---------.-.-.------

MYCNSC_NB2_237_Bta --....---------------)))))---(---((((((((.-((....((((.. .)))-)-...))..))))))))).....

------------------------------------------------------------------------------------------------------------------------------------------------------------------------------------------------
