# Supplementary material for: New miRNAs cloned from neuroblastoma
Source: BMC Genomics. 2008 Jan 29;9:52. doi: 10.1186/1471-2164-9-52 (PMC2254388; doi:10.1186/1471-2164-9-52)
Supplement: Additional file 3 — Method of comparison of biotinylated probes. Northern blot procedure for comparison of biotinylated probes is outlined. The file also contains the description of Additional File 4. [file 1471-2164-9-52-S3.doc]

# Additional File 3. Comparison of biotinylated probes for the detection of miRNAs. Comparison of biotinylated probes for the detection of miRNAs. a) 250 pg of 5’-phosphorylated sequence mimicking a cloned miRNA (CS) were resolved on 15% PAAG along with marker (M, 20nt+30nt sequences, 250 pg each). The membrane was first probed with the respective anti-miRNA probe, then striped and reprobed further with anti-marker probe. b) serial dilutions of the CONTIG_CHR_9 sequence from 150 to 10 pg (CS) were resolved on PAAG along with 30 micrograms of total tumour RNA (TS). The membrane was probed with anti-CONTIG_CHR_9 probe.
