# Supplementary figures and images for: New miRNAs cloned from neuroblastoma
Source: BMC Genomics. 2008 Jan 29;9:52. doi: 10.1186/1471-2164-9-52 (PMC2254388; doi:10.1186/1471-2164-9-52)

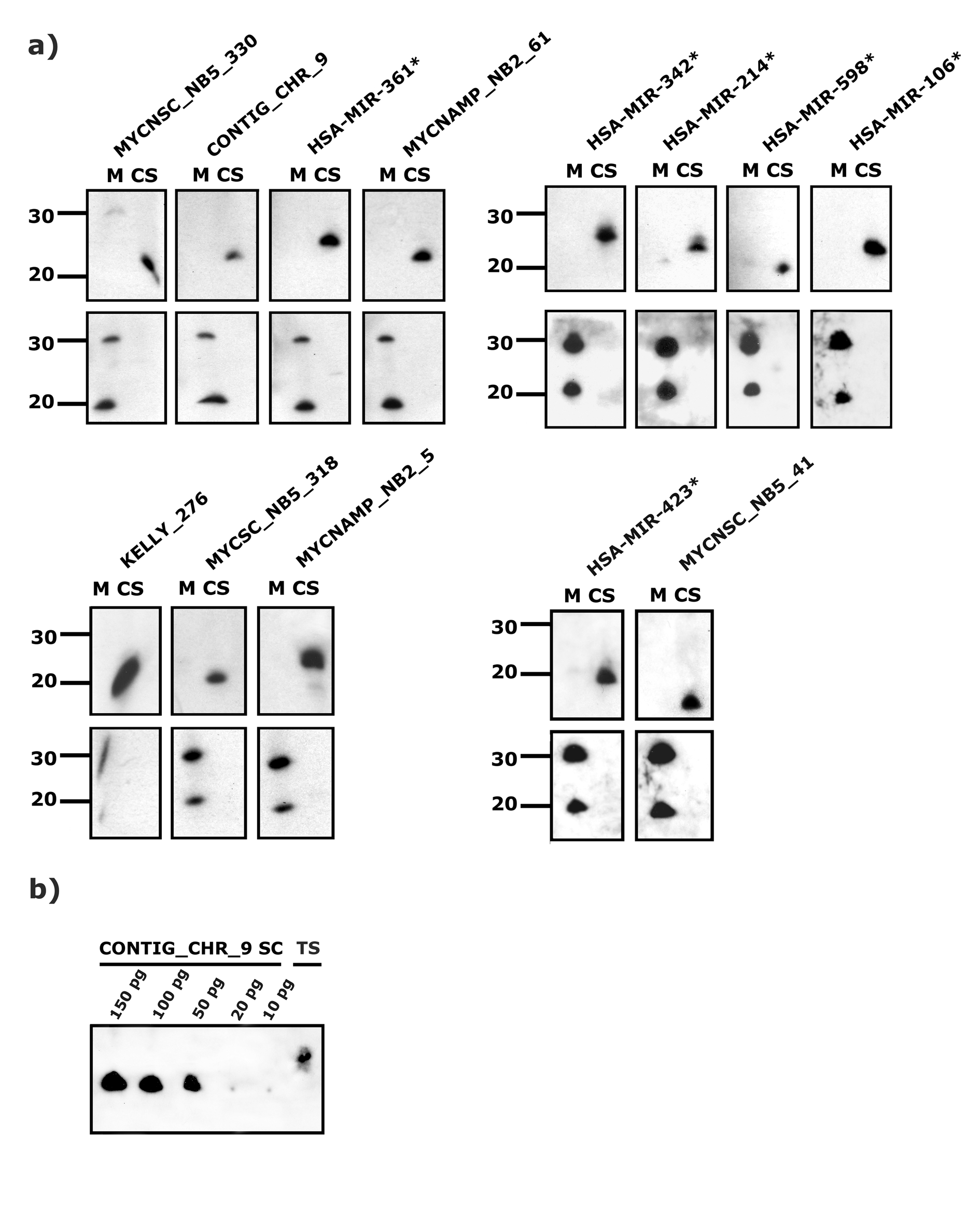

Supplement: Additional file 4 — Comparison of biotinylated probes for the detection of miRNAs. Results of Northern blotting comparing the probes used to validate novel miRNAs. [file 1471-2164-9-52-S4.tiff]
